# Development of Macrocycle Kinase Inhibitors for ALK2 Using Fibrodysplasia Ossificans Progressiva‐Derived Endothelial Cells

**DOI:** 10.1002/jbm4.10230

**Published:** 2019-10-07

**Authors:** Gonzalo Sánchez‐Duffhues, Eleanor Williams, Pascal Benderitter, Valeria Orlova, Michiel van Wijhe, Amaya Garcia de Vinuesa, Georgina Kerr, Josselin Caradec, Kirsten Lodder, Hetty C. de Boer, Marie‐José Goumans, Elisabeth M W Eekhoff, Antonio Morales‐Piga, Javier Bachiller‐Corral, Pieter Koolwijk, Alex N. Bullock, Jan Hoflack, Peter ten Dijke

**Affiliations:** ^1^ Department of Cell and Chemical Biology, Oncode Institute Leiden University Medical Center Leiden The Netherlands; ^2^ Structural Genomics Consortium University of Oxford Oxford UK; ^3^ Oncodesign SA Dijon France; ^4^ Department of Anatomy and Embryology Leiden University Medical Center Leiden The Netherlands; ^5^ Amsterdam Cardiovascular Sciences, Department of Physiology and Amsterdam Bone Center Vrije University Medical Center Amsterdam The Netherlands; ^6^ Department of Nephrology Leiden University Medical Center and the Einthoven Laboratory for Experimental Vascular Medicine Leiden The Netherlands; ^7^ Disease Research Institute, Carlos III Institute of Health (ISCIII) Madrid Spain; ^8^ Department of Rheumatology Ramon y Cajal Hospital Madrid Spain

**Keywords:** BONE MORPHOGENETIC PROTEIN, ENDOTHELIAL‐TO‐MESENCHYMAL TRANSITION, ENDOTHELIAL, FIBRODYSPLASIA OSSIFICANS PROGRESSIVA, OSTEOBLAST, TGF‐β

## Abstract

Fibrodysplasia ossificans progressiva (FOP) is an extremely rare congenital form of heterotopic ossification (HO), caused by heterozygous mutations in the activin A type I receptor (ACVR1), that encodes the bone morphogenetic protein (BMP) type I receptor ALK2. These mutations enable ALK2 to induce downstream signaling in response to activins, thereby turning them into bone‐inducing agents. To date, there is no cure for FOP. The further development of FOP patient‐derived models may contribute to the discovery of novel biomarkers and therapeutic approaches. Nevertheless, this has traditionally been a challenge, as biopsy sampling often triggers HO. We have characterized peripheral blood‐derived endothelial colony‐forming cells (ECFCs) from three independent FOP donors as a new model for FOP. FOP ECFCs are prone to undergo endothelial‐to‐mesenchymal transition and exhibit increased ALK2 downstream signaling and subsequent osteogenic differentiation upon stimulation with activin A. Moreover, we have identified a new class of small molecule macrocycles with potential activity against ALK2 kinase. Finally, using FOP ECFCs, we have selected OD36 and OD52 as potent inhibitors with excellent kinase selectivity profiles that potently antagonize mutant ALK2 signaling and osteogenic differentiation. We expect that these results will contribute to the development of novel ALK2 clinical candidates for the treatment of FOP. © 2019 The Authors. *JBMR Plus* published by Wiley Periodicals, Inc. on behalf of American Society for Bone and Mineral Research.

## Abstract 2

Endothelial colony‐forming cells (ECFCs) are isolated during routine blood tests from patients' peripheral blood. When cultured under osteogenic conditions, fibrodysplasia ossificans progressiva (FOP) endothelial colony‐forming cells resemble the aberrant activin signal transduction in FOP and can be transformed into osteogenic cells in response to BMP and activins. Using this system, we have identified OD36 and OD52 as two novel macrocyclic ALK2 kinase inhibitors with enhanced specificity for activin‐induced ALK2 downstream signaling.

## Graphical abstract

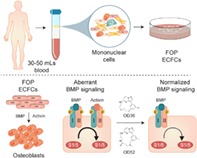

## Introduction

Heterotopic ossification (HO) is a musculoskeletal disorder characterized by bone formation at extraskeletal sites, such as soft tissues and joints. Fibrodysplasia ossificans progressiva (FOP; MIM #135100) is a devastating congenital disorder involving HO. FOP is an extremely rare disease (1 affected per 2,000,000 individuals) associated with mutations in the activin A type I receptor *(ACVR1*) gene, encoding the BMP type I receptor kinase ALK2.^1^ Based on overexpression studies, early publications suggested that the mutant ALK2 receptor becomes hypersensitive to classical BMP ligands, or even constitutively active because of the impaired interaction with the type I receptor inhibitor FKBP12,^2^ which led to the development of BMP type I receptor kinase inhibitors to prevent ALK2 downstream signaling.^3^ Two recent publications have shown that the recurrent ACVR1 c.617G > A (ALK2 R206H) mutation enables ALK2 to transduce downstream signaling through the BMP‐specific Smad1/5/8 pathway in response to activins,^4^, ^5^ a subset of ligands of the TGF‐β family, which normally induce Smad2/3 signaling via ALK4. Activins are released during inflammatory events, which may help to explain the intermittent progression of the disease, through episodes of inflammation triggered, for example, by viral infections, physical trauma, and importantly by biopsy sampling. Today, there remains no cure for FOP. Broad‐spectrum anti‐inflammatories are commonly used to ameliorate flare‐ups, but demonstrate little benefit.

Together with recent advances in the development of animal models of the disease,^4^, ^6^, ^7^, ^8^, ^9^ the establishment of patient‐derived models of FOP may contribute to study specific aspects of the disease, such as monitor disease progression or the phenotypic variability among patients with a common mutation. Despite the recent generation of induced pluripotent stem cell (iPSC) lines from FOP somatic tissues in past years,^10^, ^11^, ^12^ the establishment and maintenance of such lines remains time‐ and resource‐consuming, and is restricted to groups with expertise in iPSC technology. Therefore, new model systems making use of different cell types are progressively arising.^13^, ^14^

In this study, we have established cell cultures of circulating endothelial colony‐forming cells (ECFCs) from the peripheral blood of FOP donors, collected during routine blood tests. FOP ECFCs express the mutant *ACVR1*/ALK2 receptor and are responsive to activin A, inducing Smad1/5/8 activation and signaling. We have found that FOP ECFCs exhibit a higher BMP kinase receptor‐dependent osteogenic potential in vitro when compared with control ECFCs. Furthermore, we explored a new class of ALK2 kinase inhibitors from a small‐molecule macrocyclic platform comprised of an ATP‐mimetic scaffold and a functionalized linker. These compounds share a low molecular weight, have predictable structure–activity relationships, and drug‐like properties. Through in vitro tests in FOP ECFCs, we have identified two novel specific macrocyclic kinase inhibitors of activin‐induced mutant ALK2 signaling, with minimum effects on closely related BMP receptors. We have compared the ALK2 binding and kinase profiling between these two new molecules and the most studied BMP type I kinase receptor inhibitor, LDN‐193189, confirming their enhanced specificity for the mutant ALK2.

Altogether, we hereby introduce a new easy‐to‐establish in vitro platform to investigate FOP patient‐derived cells and its application in the identification of novel ALK2 inhibitors for FOP. These findings hold promise to help the development of future therapeutic options for this currently untreatable disease.

## Materials and Methods

### Cell lines and reagents

Mouse C_2_C_12_ myoblast cells and mouse endothelial cells 2H11 were maintained in DMEM medium (Gibco, Carlsbad, CA, USA) supplemented with 10% FBS (Invitrogen, Carlsbad, CA, USA), and penicillin/ streptomycin (Invitrogen). For 2H11 cells, the plates were pretreated with 0.1% gelatin (Sigma‐Aldrich, St Louis, MO, USA). Mouse osteoprogenitor cells KS483 were cultured in α‐MEM (Gibco) and 10% FBS (Invitrogen), and penicillin–streptomycin (Invitrogen). Human umbilical vein endothelial cells (HUVECs) were purchased from Lonza and cultured in complete EGM‐2 (Lonza, Walkerville, MD, USA) medium supplemented with 10% FBS and 0,1% penicillin–streptomycin. Mouse chondrogenic ATDC5 cells were cultured in phenol red free DMEM‐F12 (Gibco) containing 5% FBS. All of the cells were grown at 37°C in a humidified incubator with 5% CO_2_. BMP‐6 was a kind gift from Dr S Vukicevic. TGF‐β3 was provided by Dr A Hinck. Activin A was purchased from R&D Systems (Abingdon, UK). TNF‐α was purchased from Pierce (Thermo Fisher Scientific, Waltham, MA, USA).

### Blood collection and ECFCs isolation

ECFCs represent an easy source of fully functional highly proliferative ECs from humans. The isolation protocol of human ECFCs requires withdrawal of 30 to 60 mL of peripheral blood in citrate‐treated collection tubes, after obtaining written informal consent in accordance with the institutional guidelines. Usually, the first 2 mL of blood will be discarded to avoid contamination with skin fibroblasts. The blood was prediluted 1:1 with prewarmed PBS and centrifuged at 740*g* for 30 min in the presence of Ficoll Paque Plus (GE Healthcare Europe GmbH, Eindhoven, The Netherlands), to separate a fraction containing mononuclear cells (MNCs). These cells are collected and washed three times with M199 (Lonza, Verviers, Belgium) supplemented with 0.1% penicillin/streptomycin (Invitrogen, Leek, The Netherlands). Finally, the cells are resuspended in complete EGM‐2 (Lonza) supplemented with 10% platelet lysate (PL‐EGM) and 0,1% penicillin–streptomycin and seeded at density of 1.3 × 10^6^ cells/cm^2^ into 48‐well plates precoated with 3 μg/cm^2^ human collagen type I prepared according to the manufacturer's instructions (#C7624, Sigma Aldrich). After 24 hours, nonadherent cells were carefully removed and fresh medium was added to each well. From this moment, the medium is replaced every day and supernatants discarded. ECFC colonies with regular cobblestone morphology appear within 3 to 4 weeks. Isolated ECFCs are then maintained in EGM‐2 (Lonza) supplemented with 10% FBS and 0.1% penicillin/streptomycin (Invitrogen).

### Flow cytometric analysis of isolated ECFCs

Flow cytometric analysis (FACS) of isolated ECFCs was performed as previously described.^15^ Briefly, cells were dissociated using 1X TrypLE select (Invitrogen) and washed once with the FACs buffer with 10% FBS, followed by an additional wash with FACs buffer. A list of the antibodies used can be found as Online Supplemental data. The samples were analyzed with the MACSQuant VYB (Miltenyi, Bergisch Gladbach, Germany) with the following instrument settings: Blue/488 FITC, A488: 525/50; Yellow/561 PE: 586/15, APC: 661/20, APC‐Cy7: 750 LP.

### Genotype analysis

There were 100 ng of DNA subjected to PCR to amplify the exon 4 of *ACVR1/ALK2* using hALK2ex4FW (CCAGTCCTTCTTCCTTCTTCC) and hALK2ex4RV (AGCAGATTTTCCAAGTTCCATC), as reported previously.^1^ The PCR product was separated in a 1% agarose gel and the 350‐bp fragment was cut and purified using Wizard (Promega, San Luis Obispo, CA, USA). Samples were submitted to Sanger sequencing using both hALK2ex4FW and hALK2ex4RV oligonucleotides.

### Quantitative real‐time RT‐PCR (qPCR)

Total RNA extraction was performed using NucleoSpin RNA II (Machery Nagel, Düren, Germany). There were 500 ng of RNA retro‐transcribed using RevertAid First Strand cDNA Synthesis Kits (Fisher Scientific, Landsmeer, The Netherlands), and real‐time reverse transcription‐PCR experiments were performed using SYBR Green (Bio‐Rad, Veenendaal, The Netherlands) and a Bio‐Rad CFX Connect device. A list of the oligonucleotides used can be found as online supplemental data.

### Mineralization assays

For mineralization assays, 5 × 10^4^ ECs were seeded into 48‐well plates and incubated in osteogenic medium containing 10^−8^M/L dexamethasone, 0.2mM/L ascorbic acid, and 10mM/L β‐glycerolphosphate in the presence of BMP/ TGF‐β ligands for 28 days. The medium was refreshed every 4 days. Afterwards, cells were washed twice with PBS and fixed with 3.7% formaldehyde for 5 min. Next, cells were washed twice with distilled water; measurement of calcium deposition was performed by Alizarin Red solution (ARS) staining, as previously described.^16^ Precipitates, originated from three independent ARS assays, were dissolved using 10% cetylpyridinium chloride, and absorbance was measured at 570 nm. Representative pictures were obtained using a Leica DMIL LED microscope (Leica, Wetzlar, Germany) with 10× magnification.

### Chondrogenic differentiation assays and adenovirus transduction

Forty‐eight hours before starting the micromass assay, the ATDC5 cells were transduced with the same titer of adenoviral particles in the presence of Polybrene (4 mg/mL), encoding for either the ALK2wt‐HA or ALK2R206H‐HA (previously described^2^). Briefly, ATDC5 cells were trypsinized and washed once with PBS. There were 3 × 10^5^ cells counted per micromass, and resuspended in 10 μL of culture medium. Very carefully, 100‐μL drops were deposited in the center of the well in a 24‐wells plate and placed in the incubator for 2 hours. Next 500 μL of DMEM‐F12 5% FBS containing 1X ITS (Gibco) were carefully added to the wells. After 24 hours, the medium was replaced by DMEM‐F12 5% FBS containing 1X ITS, supplemented with BMP‐6 or activin A (100 ng/mL), and DMSO, LDN‐193189, OD36, or OD52 (0.5μM). The cells were incubated for 21 days before further analysis, refreshing the medium every 5 days. To stain the pellets, cells were fixed for 15 min in 500 μL of fixative solution (30% EtOH, 0.4% PFA, and 4% acetic acid). Next, the fixative solution was removed and the pellets were incubated overnight at 37°C in Alcian Blue staining solution (0.05% Alcian Blue staining solution in 75% EtOH:0.1M HCl [4:1]). Finally, the cells were washed and pictures acquired using a Leica DMIL LED microscope with 10× magnification. Subsequently, the staining was solubilized in 250 μL of 6 guanidine hydrochloride (Sigma‐Aldrich) and quantification was performed by absorbance at 595 nm.

### Statistical analysis

Student's *t* test was used for statistical analysis and *p* < 0.05 was considered to be statistically significant. All experiments were performed at least in triplicate, unless indicated.

## Results

### Establishment and characterization of FOP ECFCs

**Figure 1:**
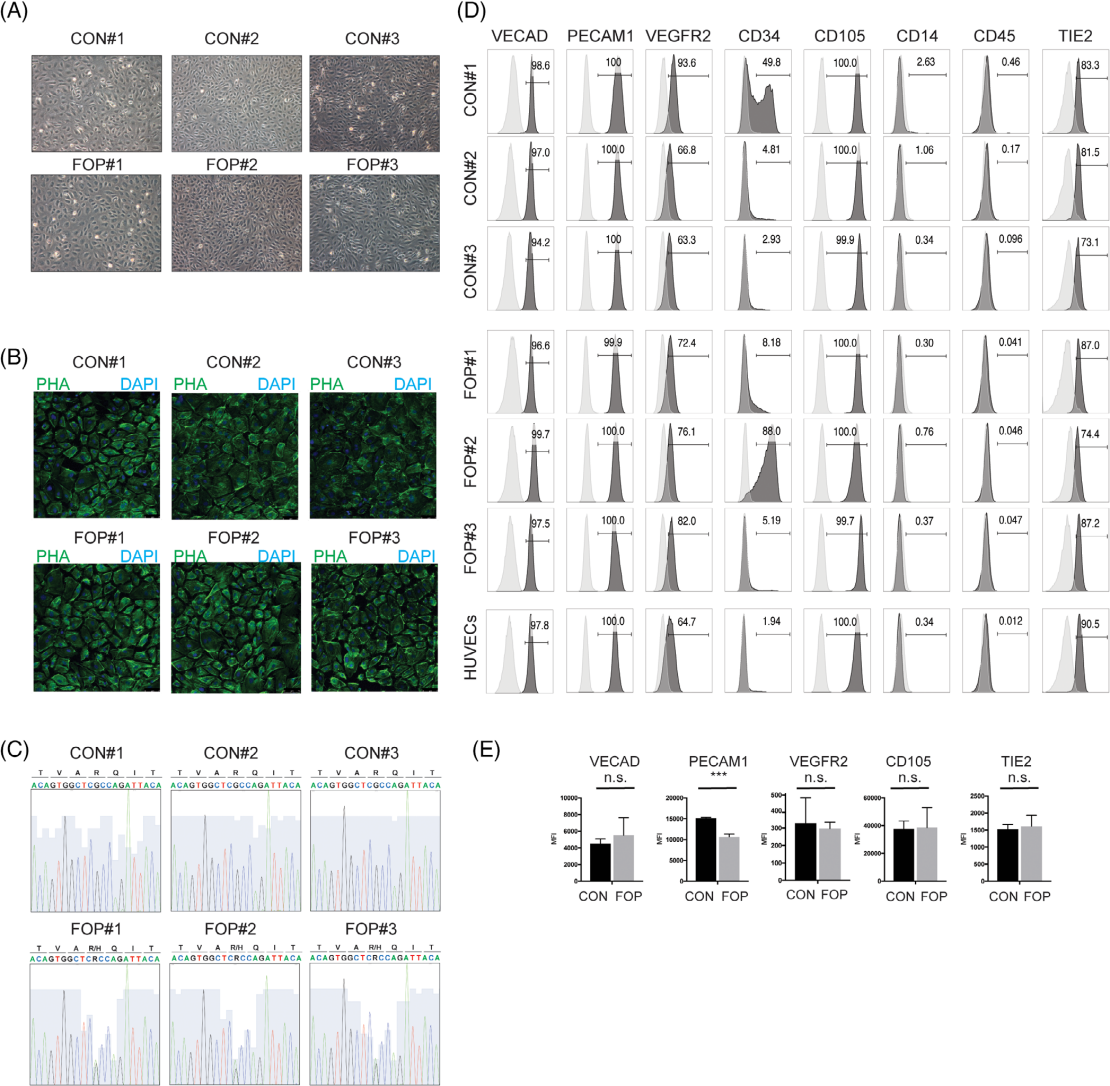
Establishment of control and fibrodysplasia ossificans progressiva (FOP) endothelial colony‐forming cell (ECFC) cultures. (*A*) Bright field representative images of three independent confluent control (CON#1, CON#2 and CON#3) and FOP (FOP#1, FOP#2 and FOP#3) ECFCs. PHA: phalloidin. (*B*) Cell morphology in control and FOP ECFCs is shown upon phalloidin staining. Nuclei are visualized in blue (4,6‐diamidino‐2‐phenylindole). (*C*) Sanger sequencing of *ACVR1/ALK2* exon 4 shows the classical heterozygous (c.617G > A; R206H) mutation in FOP ECFCs. (*D*) Flow cytometry analysis of EC (Ve‐Cadherin, PECAM‐1, VEGFR2, CD105, and Tie2), immune cell (CD14 and CD45), and hematopoietic (CD34) markers in control and FOP ECFCs. (*E*) Mean fluorescent intensity of three independent control and FOP ECFCs. ***p < 0.001.

**Figure 2:**
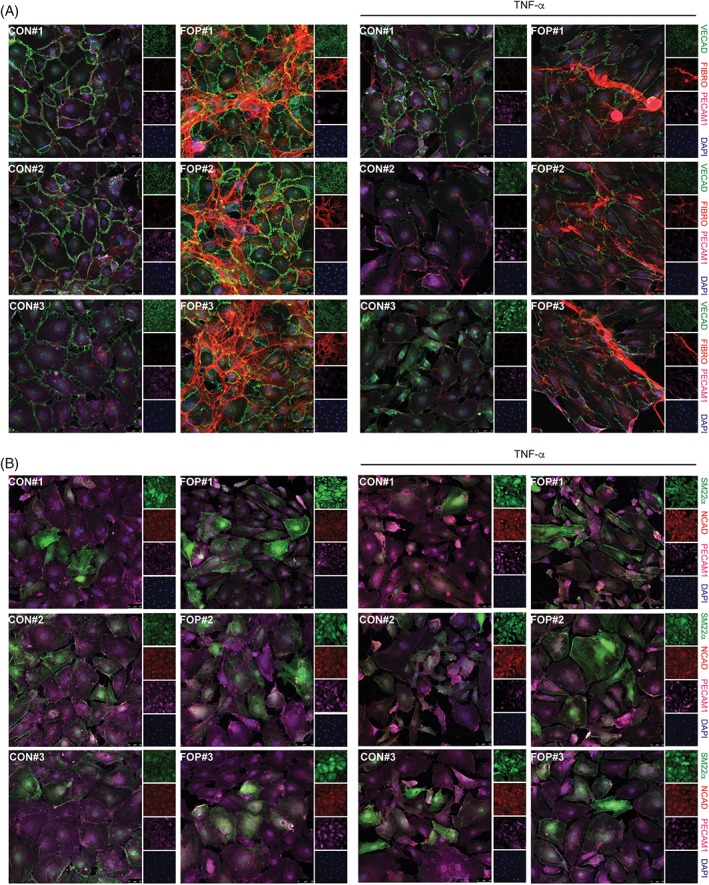
Fibrodysplasia ossificans progressiva (FOP) endothelial colony‐forming cells (ECFCs) are prone to undergo endothelial‐to‐mesenchymal transition (EndMT) in response to TNF‐α. Control (CON#1, CON#2 and CON#3) and FOP (FOP#1, FOP#2, and FOP#3) ECFCs were stimulated with TNF‐α (10 ng/mL) for 24 hours prior to immunofluorescent labeling for either Ve‐cadherin (VECAD, green), fibronectin (FIBRO, red), and PECAM1 (PECAM, magenta) (*A*) or Transgelin (SM22α, green), N‐cadherin (NCAD, red), and PECAM1 (PECAM, magenta) (*B*). Nuclei are visualized in blue (4,6‐diamidino‐2‐phenylindole). Representative confocal images are shown.

We used a previously reported minimally invasive protocol^17^ to isolate endothelial‐colony‐forming cells (ECFCs) from the peripheral blood of FOP patients diagnosed with the FOP classical variant (see Supplementary Table S1 for donor characteristics). Confluent ECFCs under the microscope exhibited an endothelial‐like appearance (Fig. 1 *A*), with apparently no morphological differences between the control and FOP colonies as seen upon cytoskeleton F‐actin staining (Fig. 1 *B*). Next, we confirmed the presence of the classical heterozygous *ACVR1/ALK2* (c.617G > A; R206H) mutation in our three FOP donors (Fig. 1 *C*). To substantiate the endothelial characteristics of the cultures, we determined by flow cytometry analysis (FACS) the surface expression of the endothelial‐specific proteins vascular endothelial (VE) cadherin; platelet endothelial cell adhesion molecule (PECAM‐) 1; vascular endothelial growth factor receptor‐ (VEGFR‐) 2, CD105, and Tie‐2; the hematopoietic marker CD34; and the immune cell markers CD14 and CD45 (Fig. 1 *D*). Both control and FOP ECFCs were negative for the immune markers CD14 and CD45, and expressed similar levels of endothelial proteins as human umbilical vein endothelial cells (HUVECs), used as a reference. Of note, control and FOP ECFCs express PDGFRα (Supplementary Fig. S1 *A*), which has been shown to be expressed by fibroadipogenic progenitor cells in mouse models of FOP.^8^ The mean PECAM‐1 expression was significantly lower in FOP ECFCs, compared with controls (Fig. 1 *E*), which is suggestive of a process named endothelial‐to‐mesenchymal transition (EndMT), by which ECs lose their phenotypic characteristics and progressively become fibroblast‐like cells, susceptible to further differentiation into chondrocytes or osteoblasts.^(13)^ Endothelial cells overexpressing the FOP mutant ALK2 receptor are prone to undergo EndMT in response to TGF‐β and BMP ligands.^18^ In addition, EndMT has been unveiled as a key phenomenon for proper cardiovascular development and evidence for its contribution to the onset and progression of postnatal cardiovascular disease is accumulating.^19^ To investigate whether FOP ECFCs may be sensitized to undergo EndMT and become fibroblast‐like cells, we performed immunofluorescent labeling (Fig. 2 *A*,*B*) and qPCR analysis (Supplementary Fig. S1 *B*) for the endothelial markers *PECAM1* and *CDH5* (encoding for Ve‐cadherin) and the mesenchymal markers fibronectin (*FN1*), SM22α (*TAGLN*), and N‐cadherin (*CDH2*). Furthermore, because EndMT is induced in response to inflammation, we investigated whether control and FOP ECFCs display a mesenchymal phenotype in response to the proinflammatory cytokine tumor necrosis factor (TNF)‐α. FOP ECFCs did express notoriously higher levels of the mesenchymal markers fibronectin (*FN1*) and SM22α (*TAGLN*) than control ECFCs, whereas there were no evident differences in Ve‐cadherin expression. Moreover, *FN1* and *CDH2* were significantly upregulated, whereas *PECAM1* was downregulated in control and disease cell lines in response to TNF‐α. Finally, whereas we failed to amplify the tendon‐specific HO progenitor gene marker *SCX* by qPCR (data not shown), we found that *MX1* expression was induced in control and FOP ECFCs in response to inflammation, suggesting that FOP ECFCs may resemble the phenotype of muscle resident injury‐induced HO progenitor cells in humans^20^ (Supplementary Fig. S1 *C*).

### FOP ECFCs exhibit an enhanced osteogenic response to activin A

**Figure 3:**
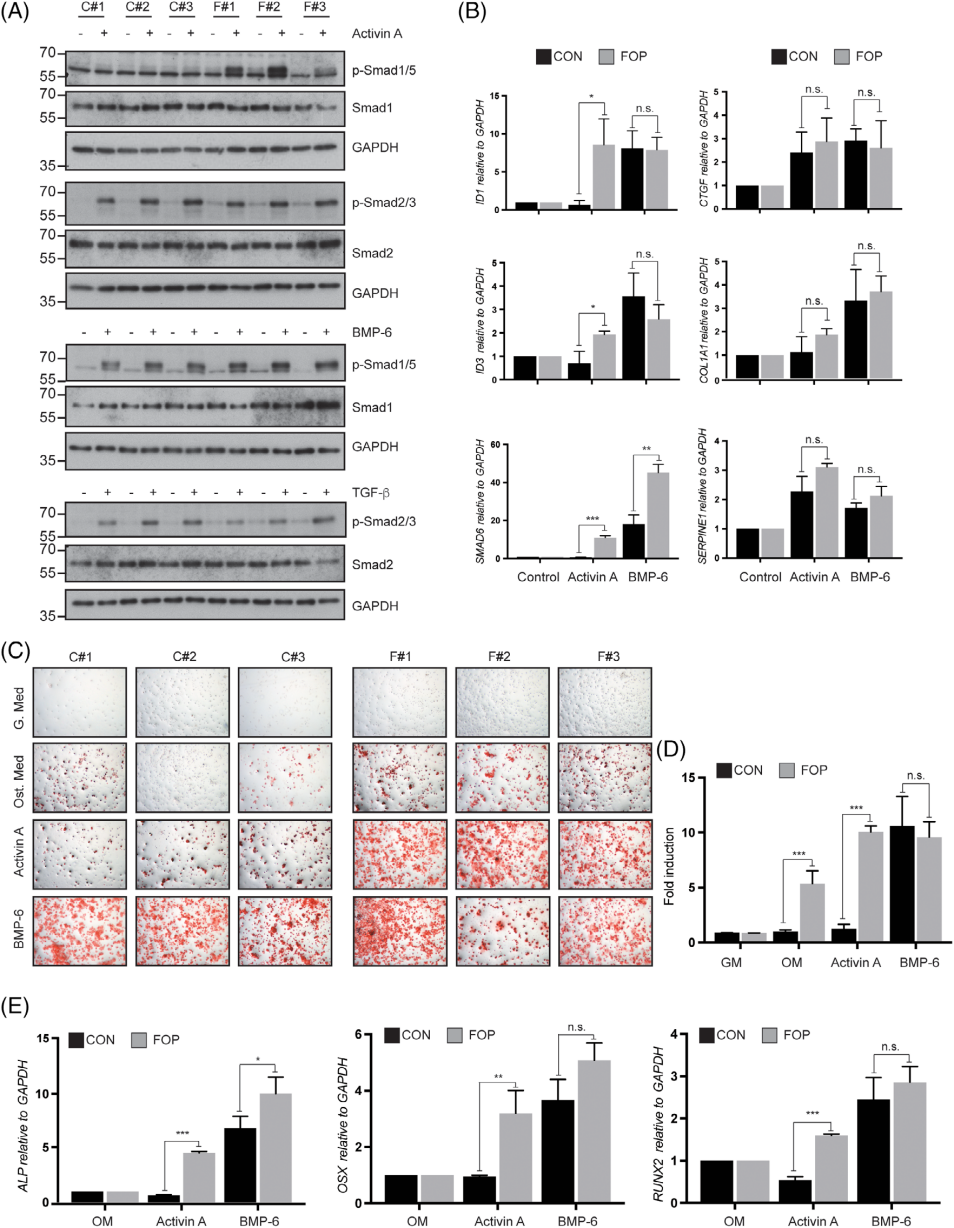
Fibrodysplasia ossificans progressiva (FOP) endothelial colony‐forming cells (ECFCs) activate Smad1/5 signaling in response to activin A, and exhibit an enhanced osteogenic potential. (*A*) Phospho‐Smad1/5, phospho‐Smad2/3 and total Smads Western blot analysis in control and FOP ECFCs stimulated after serum starvation with either activin A (50 ng/mL), BMP‐6 (50 ng/mL), or TGF‐β (5 ng/mL) for 45 min. (*B*) Expression of the BMP‐target genes *ID1*, *ID3*, and *Smad6* and the TGF‐β target genes *CTGF*, *Col1a1*, and *Pai‐1* in control and FOP ECFCs stimulated with either activin A (50 ng/mL) or BMP‐6 (50 ng/mL) for 3 hours. (*C*) Alizarin Red solution staining in control and FOP ECFCs incubated with either activin A (50 ng/mL) or BMP‐6 (50 ng/mL) under osteogenic conditions. One representative image is shown per clone. (*D*) Quantification of (*C*) by absorbance upon solubilization of calcium deposits in cetylpyridinium. GM: growth medium; OM: osteogenic medium. (*E*) Expression of the osteogenic genes alkaline phosphatase (*ALP*), osterix (*Osx*), and *Runx2* in control and FOP ECFCs incubated under osteogenic conditions with or activin A or BMP‐6 (50 ng/mL). *p < 0.05, **p < 0.01, ***p < 0.001.

The recent discovery, concerning the neofunction of ALK2 R206H enabling the receptor to respond to activin, has highlighted a novel mechanism by which mutant ALK2 induces HO in FOP in an ALK2 kinase activity‐dependent manner.^4^ To validate ECFCs as a surrogate model for FOP, we first confirmed the expression of *ACVR1* by qPCR (Supplementary Fig. S2). Analysis of the expression of the TGF‐β and BMP receptors and coreceptors revealed a significant increase in the mRNA levels of *ACVR1L*, *ACVR1B*, *BMPR2*, and *TDGF1* in FOP‐derived ECFCs. Next, we analyzed the effect of recombinant activin A administration on canonical Smad 1/5 signaling. As shown in Fig. 3 *A*, exclusively FOP ECFCs responded to activin A by inducing the phosphorylation of Smad 1/5 (p‐Smad1/5), and the expression of *ID‐1*, *ID‐3*, and *Smad6*, which are downstream of ALK2‐Smad1/5 signaling (Fig. 3 *B*). Of note, control and FOP ECFCs displayed a similar Smad2/3 phosphorylation (p‐Smad2/3) in response to activin A, suggesting that the classical ALK4‐activin A pathway remains unaffected in FOP ECFCs. Furthermore, p‐Smad2/3 was similarly induced by TGF‐β in control and FOP cells. Accordingly, we did not find significant differences in the activation of the Smad3 target genes connective tissue growth factor (*CTGF*), collagen‐1‐alpha‐1 (*Col1a1*), or serpine‐1 (*Pai‐1*) (Fig. 3 *B*).

ECs overexpressing the FOP mutant *ACVR1*/ALK2 receptor have been reported to undergo EndMT in response to BMP‐4 and TGF‐β, and to differentiate into osteoblasts.^18^ Based on those results, we established an in vitro model to differentiate ECFCs into osteogenic cells. We incubated control and FOP ECFCs in osteogenic medium for 4 weeks and visualized the deposition of calcified matrix by ARS staining (Fig. 3 *C*). Stained deposits were subsequently dissolved and quantified by absorbance (Fig. 3 *D*). FOP ECFCs exhibited an enhanced staining under basal osteogenic conditions, whereas cells incubated in normal growth medium were not stained by ARS. Interestingly, BMP‐6 induced the formation of calcium deposits at a similar level in control and FOP ECFCs, whereas activin A induced significantly more ARS staining in FOP ECFCs. These results were supported by the analysis of the expression of the osteoblast‐specific genes alkaline phosphatase (*ALP*), osterix (*Osx*), and *Runx2* by qPCR (Fig. 3 *E*). As predicted from previous work,^4^ both activin A‐induced Smad1/5 phosphorylation and subsequent osteogenic differentiation were inhibited upon treatment with the ALK2 kinase inhibitor LDN‐193189 (Fig. 6 *D*–*F*), demonstrating a requirement for the kinase activity of ALK2 for these responses.

### Identification of two novel macrocyclic inhibitors OD36 and OD52 with potent binding to the ALK2 kinase ATP pocket

**Figure 4:**
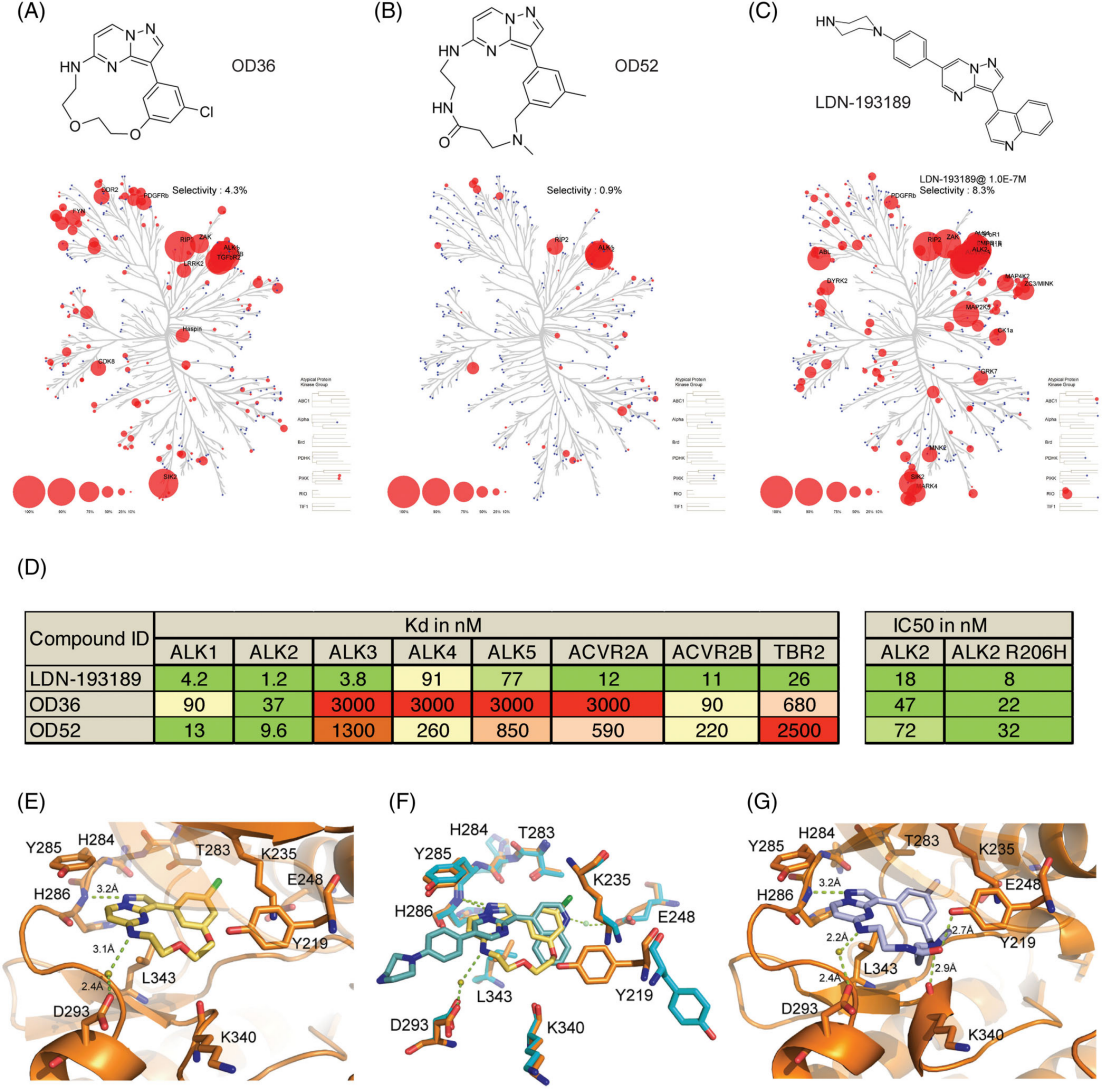
ALK2 binding and kinase profile of OD36 and OD52. The activity of a panel of 366–468 kinases was tested in the presence of 100nM of OD36 (*A*) or OD52 (*B*) and compared with LDN‐193189 (*C*), adapted and used with permission from *Science* (sciencemag.org) and Cell Signalling Technology (Danvers, MA, USA). Circle size shows potency. (*D*) Summary of the affinity (Kd) of OD36, OD52, and LDN‐193189 for the BMP and TGF‐β type I and type II kinase receptors, and the IC50 of the three ALK2 antagonists for the WT and R206H ALK2 receptor. (*E*) Cocrystal structure of ALK2 and OD36 (2.56 Å) and (*F*) superposition with LDN‐193189. (*G*) Model of OD52‐ALK2 interaction based on shape complementary as predicted by modulus of elasticity (MOE).

The dependence of the pathological signaling in FOP on ALK2 kinase activity has stimulated the development of the first small molecule ALK2 inhibitors.^3^, ^21^, ^22^ We decided to use an alternative approach to develop a novel class of molecules antagonizing ALK2 kinase activity. The nanocyclix platform is a chemistry‐based platform of small‐molecular‐weight macrocycles, designed as ATP‐competitive type 1 inhibitors. Their unique tridimensional shape is thought to be at the origin of their affinity and selectivity. Among other inhibitors with affinity for receptor interacting serine/threonine kinase 2 (RIPK2),^23^ we identified a chemical series with high affinities against ALK2, while displaying interesting selectivity (including OD36). Further characterization highlighted a good selectivity against the other members of the BMP receptor family, as seen in Supplementary Table S2. A first round of optimization, consisting of the modification of the linker focused on the improvement of selectivity, led to the identification of the lead compound OD52, displaying similar affinities against ALK2, while improving overall kinase selectivity and significantly improving solubility (Supplementary Tables S2 and S3). As Fig. 4 *A–C* shows, whereas LDN‐193189 showed a poor selectivity, inhibiting over 50% of the activity in 8.3% of all kinase activities tested, OD36 and OD52, in particular, displayed an improved selectivity (only 4.3% and 0.9% of tested kinases were inhibited). This was further validated by the determination of the *K*_D_ values of OD36 and OD52 against kinase receptors of the TGF‐β and BMP family (Fig. 4 *D*). Importantly, OD36 and OD52 both showed ALK2‐directed activity with *K*_D_ values of 37nM and 9.6nM, respectively (Fig. 4 *D*). In addition, we determined the in vitro IC50 values against the WT and mutant R206H receptor kinase domains, which showed an enhanced activity for the mutant receptor. Finally, we solved the crystal structure of OD36 in complex with ALK2 refined at 2.56 Å resolution (PDB ref. 5OY6) (Fig. 4 *E*, Supplementary Table S4). Comparison of the binding modes of OD36 and LDN‐193189 in the ALK2 ATP pocket (Fig. 4 *F*) revealed a common positioning of the pyrazolo[1,5‐a]pyrimidine groups, allowing a single hydrogen bond to the ALK2 hinge residue His286, while the phenylchloride moiety of OD36 occupied the back pocket similar to the 4‐quinoline of LDN‐193189. The macrocyclic linker in OD36 also afforded an additional water‐mediated hydrogen bond to Asp293. In silico modeling of OD52 based on the OD36 cocrystal revealed a conserved binding mode, but predicted two additional hydrogen bonds from the variable linker to Tyr219 and Lys340, respectively (Fig. 4 *G*).

### OD36 and OD52 represent a new class of BMP receptor inhibitors with enhanced specificity for ALK2 kinase activity

**Figure 5:**
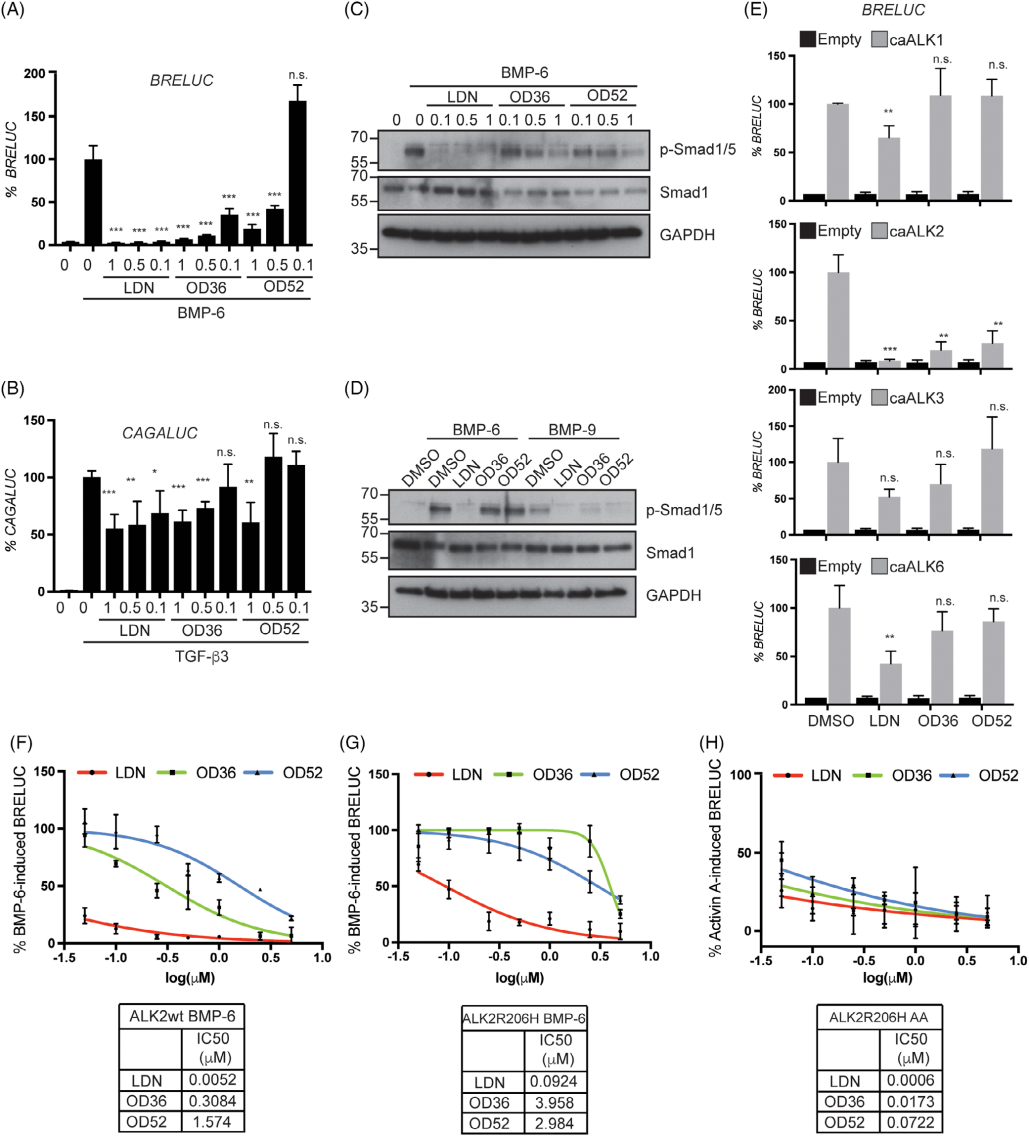
The macrocyclic compounds OD36 and OD52 efficiently inhibit activin A‐induced ALK2 R206H downstream signaling. (*A*) BMP BRE‐LUC reporter assay performed in KS483 preincubated with LDN‐193189, OD36, or OD52 at the indicated concentrations, followed by stimulation with BMP‐6 (50 ng/mL) for 16 hours. (*B*) TGF‐β CAGA‐LUC reporter assay performed in KS483 cells preincubated with LDN‐193189, OD36, or OD52 at the indicated concentrations, followed by stimulation with TGF‐β (5 ng/mL) for 16 hours. (*C*) Analysis of BMP‐6‐induced Smad1/5 phosphorylation and total Smad1 expression by Western blot in KS483 cells, in the presence of LDN‐193189, OD36, or OD52. (*D*) Western blot of 2H11 endothelial cells stimulated with either BMP‐6 (50 ng/mL) or BMP‐9 (1 ng/mL), upon preincubation with LDN‐193189, OD36, or OD52 at 0.5μM. (*E*) Cotransfections of BRE‐LUC and constitutive active (ca) forms of the ALK1/2/3/6 receptors in C_2_C_12_ cells. Compounds were added for 16 hours at 0.5μM before luciferase activity was measured. Dose‐dependent response of LDN‐193189 (LDN), OD36, or OD52 in C_2_C_12_ cells cotransfected with the BRE‐LUC reporter plasmid and the WT (*F*) or R206H ALK2 receptor (*G*, *H*). Upon 30‐min preincubation with the indicated compounds, the cells were stimulated with BMP‐6 (*F*, *G*) or activin a (*H*) (50 ng/mL) for 16 hours. IC50 is indicated. *p < 0.05, **p < 0.01, ***p < 0.001.

We next tested OD36 and OD52 in traditional cell models to assess BMP activity, using murine preosteoblast KS483 cells transfected with the BMP responsive element‐ (BRE‐) driven transcriptional luciferase reporter construct BRE‐LUC.^24^ Stimulation of the reporter activity using BMP‐6, which signals through ALK2, ALK3, and ALK6, was inhibited in a dose‐dependent manner in the submicromolar range by the macrocyclic inhibitors OD36 and OD52, although with less potency than the reference compound LDN‐193189 (Fig. 5 *A*). Using the TGF‐β/Smad3 reporter construct CAGA‐LUC,^25^ we observed that OD36 and OD52 had significantly less inhibitory effect on TGF‐β‐induced gene transcription than LDN‐193189 (Fig. 5 *B*), consistent with the recombinant kinase selectivity profiling. Congruently, OD36 and OD52 efficiently inhibited BMP‐6‐ induced p‐Smad1/5 in KS483 cells (Fig. 5 *C*). After confirming by ligand‐binding affinity assays with radiolabeled BMP‐9 that BMP‐9 mainly signals through ALK2 in 2H11 ECs (Supplementary Fig. S3), we evaluated the activity of OD36 and OD52 to specifically inhibit ALK2‐induced signaling by stimulating these cells with either BMP‐6 or BMP‐9. As Fig. 5 *D* shows, LDN‐193189 efficiently blocked p‐Smad1/5 in response to both BMP‐6 and BMP‐9, whereas OD36 and OD52 only inhibited BMP‐9‐induced p‐Smad1/5. Next, we cotransfected C_2_C_12_ murine myofibroblast cells with constitutive active (ca) constructs of the BMP type I receptors ALK1, ALK2, ALK3, and ALK6 and the BRE‐LUC construct.^26^ Interestingly, whereas LDN‐193189 significantly inhibited caALK1/2/6 at 0.5μM, OD36 and OD52 exclusively inhibited ALK2 (Fig. 5 *E*). Finally, to compare the IC50 of OD36, OD52, and LDN in living cells, we performed a reporter assay in C_2_C_12_ cells cotransfected with the BMP reporter construct BRELUC and either the WT or the mutant ALK2 R206H. Noteworthy, when testing different concentrations (0, 0.05, 0.1, 0.25, 0.5, 1, 2.5, 5μM) of the indicated compounds, the IC50 of LDN was comparable to that of OD36 and OD52 in cells stimulated with activin A in the presence of the mutant receptor (Fig. 5 *H*), which is not the case in cells stimulated with BMP‐6, irrespective of overexpressing the WT or the mutant ALK2 (Fig. 5 *F*,*G*). Altogether, this demonstrates the specificity of OD36 and OD52 for ALK2.

### OD36 and OD52 inhibit activin A‐induced ALK2 R206H osteogenic and chondrogenic differentiation

**Figure 6:**
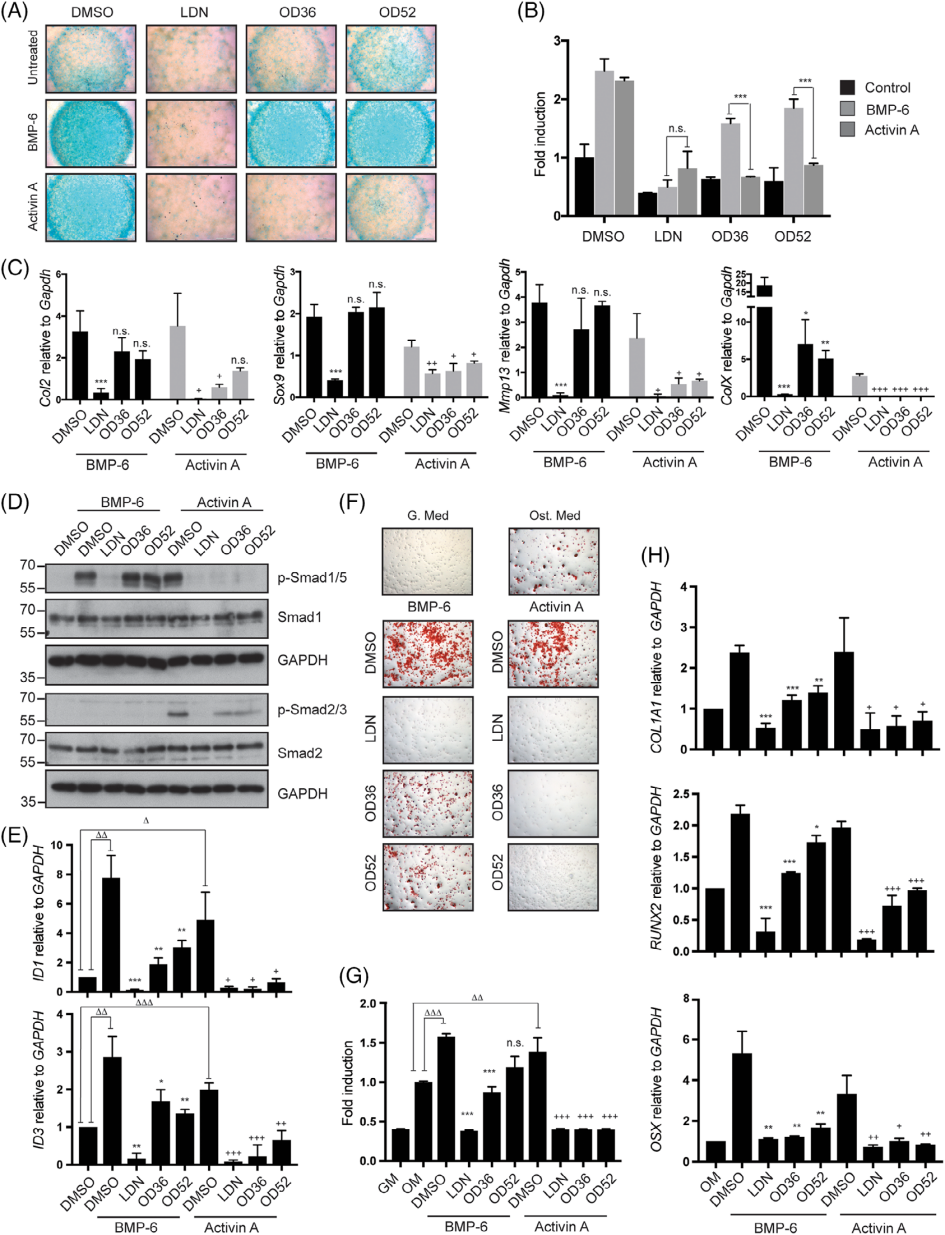
The macrocyclic compounds OD36 and OD52 efficiently inhibit activin A‐induced chondrogenic and osteogenic differentiation in in vitro models of Fibrodysplasia ossificans progressiva (FOP). (*A*) Chondrogenic differentiation micromass assay in ATDC5 cells overexpressing ALK2 R206H. Cells were incubated for 21 days in chondrogenic medium containing BMP‐6 or activin A (50 ng/mL) and LDN‐193189, OD36, or OD52 at 0.5μM, prior to Alcian Blue staining. (*B*) Quantification of Fig. 6 *A* by absorbance, after solubilization with guanidine hydrochloride. (*C*) Chondrogenic gene expression analysis of collagen 2 (*Col2*), *Sox9*, Matrix metalloproteinase 13 (*Mmp13*), and collagen X (*Col X*) in ATDC5 cells corresponding to (*A*). (*D*) Western blot of a representative clone of FOP endothelial colony‐forming cells (ECFCs) incubated with LDN‐193189, OD36, or OD52 (0.5μM) prior to stimulation with BMP‐6 (50 ng/mL) or activin A (50 ng/mL). (*E*) Expression of the BMP target genes *ID1* and *ID3* by qPCR in FOP ECFCs stimulated with BMP‐6 (50 ng/mL) or activin A (50 ng/mL) in the presence of the indicated compounds at 0.5μM. (*F*) Alizarin Red solution staining on a representative clone of FOP ECFCs incubated under osteogenic conditions with BMP‐6 (50 ng/mL) or activin A (50 ng/mL) and the indicated compounds. (*G*) Quantification of Fig. 6 *F* by absorbance, after solubilization with cetylpyridinium. (*H*) Osteogenic genes Collagen 1 alpha 1 (*Col1a1*), *Runx2*, and osterix (*Osx*) expression in a representative clone of FOP ECFCs incubated under osteogenic conditions with BMP‐6 (50 ng/mL) or activin A (50 ng/mL) and LDN‐193189, OD36, or OD52 (0.5 μM). *p < 0.05, **p < 0.01, ***p < 0.001.

To test the potential of OD36 and OD52 to block endochondral ossification in FOP, we used ATDC5 chondrogenic progenitors as a previously validated model system.^27^ Using adenoviruses, we transduced ATDC5 with vectors encoding for the WT or the mutant R206H ALK2 receptor. As shown in Supplementary Fig. S4, ATDC5‐ALK2 R206H exhibited a significant increase in Alcian Blue staining with respect to ATDC5‐ALK2 WT in response to activin A stimulation, which was further supported by the expression analysis of the chondrocyte genes *Sox9*, *Mmp13*, and *ColX*. In this model, LDN (0.5μM) indistinctly inhibited BMP‐6 and activin A‐induced chondrogenic differentiation. OD36 and OD52 (0.5μM), however, exhibited a very potent inhibitory effect on activin A‐induced chondrogenic differentiation, while showing a minor response to BMP‐6 (Fig. 6 *A*–*C*). Finally, we determined the effect of these two compounds in FOP ECFCs subjected to osteogenic differentiation, therefore resembling a FOP human genetic background. As shown in Fig. 6 *D* and 6 *E*, preincubation of FOP ECFCs with OD36 and OD52 completely prevented the activation of Smad1/5 and gene targets *ID‐1* and *ID‐3* in response to activin A, whereas they were less effective on BMP‐6 induced downstream signaling. Furthermore, OD36 and OD52 efficiently blocked activin A‐induced osteogenic differentiation of FOP ECFCs, but they showed a modest effect upon stimulation with BMP‐6 (Fig. 6 *F*–*H*). This indicates that specific targeting of activin A‐ALK2 FOP‐induced signaling might be sufficient to prevent osteogenic differentiation in FOP. In summary, we have introduced ECFCs from FOP donors as an in vitro model of the disease, recapitulating the aberrant response of FOP cells to activin, and unveiled macrocyclization technology as a novel approach to generated ALK2 kinase inhibitors with improved specificity. We hope that our results will provide novel tools to encourage the development of validated and safe therapeutic approaches for FOP.

## Discussion

In this study, we introduced ECFCs as a novel in vitro model for FOP and used it to identify novel specific inhibitors of activin A‐induced osteogenic differentiation. FOP ECFCs recapitulate the pathogenic mechanisms underlying FOP, in particular, the induction of Smad1/5‐mediated signaling by activin A, consistent with recent publications.^4^, ^5^ Noteworthy, such ALK2 neofunction in FOP ECFCs is sensitive to inhibitors of ALK2. We have developed a new class of ALK2 kinase domain small‐molecule antagonists, consisting of macrocyclic compounds exhibiting an improved selectivity for ALK2 against closely related TGF‐β and BMP kinase receptors. OD36 and OD52 bind to ALK2 through interaction with the kinase hinge residue His286, but explore a larger area within the ATP pocket than LDN‐193189 because of their macrocycle linkers, which offer an improved shape complementarity contributing to their high selectivity. Finally, using FOP‐derived ECFCs, as well as traditional in vitro model systems for BMP and TGF‐β, we have demonstrated that OD36 and OD52 specifically inhibit ALK2 downstream signaling with enhanced selectivity over more promiscuous BMP signaling pathways inhibitors, therefore blocking activin A‐ALK2 R206H‐induced osteogenic and chondrogenic differentiation.

After the discovery of the *ACVR1* mutation associated with FOP by sequencing different patient‐derived biopsies (ie, lymphoblastic lines, buccal swabs, or blood samples),^1^ efforts have been focused to understand the aberrant mechanisms triggered in patients, resulting in misregulated endochondral ossification. The establishment of patient‐derived models of FOP has been traditionally impaired by the induction of HO upon tissue biopsy sampling. Therefore, studies were often based on overexpression strategies that may result in experimental artifacts. Other approaches have been pursued, including the collection of stem cells from human exfoliated deciduous teeth (SHED),^28^ or fibroblasts isolated from either the skin^14^ or periodontal ligaments.^13^ In parallel, different groups have engineered a number of FOP animal models. To overcome the embryonic lethality caused by the embryonic expression of ALK2 R206H, the recently developed models express *ACVR1* c.617G > A postnatally, or in a tissue‐specific manner. Such mouse models have allowed the preclinical evaluation of different potential treatments for FOP. In addition, in FOP mice different cell populations have been described to mediate HO in a cell autonomous manner, generally termed fibroadipogenic cells (FAPs).^8^, ^20^ None of the human‐derived models aforementioned exhibit a phenotype similar to that of FAPs.^8^, ^18^, ^20^ Although iPSCs have facilitated the establishment of patient‐derived cell models, they remain technically challenging, and time‐ and resource‐consuming. Furthermore, in the particular case of endothelial cells, whether iPSC‐derived endothelial cells exactly recapitulate the existent endothelial cells in FOP patients is not known. For example, FOP iPSC‐derived endothelial cells do not proliferate normally, and interestingly, do not induce ALK2 downstream signaling in response to activin A,^11^ suggesting that iPSC‐endothelial cell differentiation protocols may need to be optimized to obtain mature FOP endothelial cells. In comparison with existing methods, ECFCs are easily accessible and not invasive when combined with routine blood withdraws. ECFCs are isolated following a protocol already used for the isolation of immune cells from peripheral blood, which is minimally modified to favor ECFC colony proliferation and can be cultured for multiple passages. ECFCs have been previously used as surrogates to study vascular diseases (reviewed in^29^). Interestingly, a number of vascular comorbidities have been reported in the few existing manuscripts containing patient‐derived tissues, including ventricular dysfunction with cardiac fibrosis^30^ and pulmonary hypertension caused by a decreased pulmonary vascular bed.^31^ Both endothelial dysfunction and, in particular, EndMT have been demonstrated to contribute such adult pathologies.^32^, ^33^ Therefore, we anticipate that ECFCs may be interesting to investigate these secondary conditions in FOP. In addition, more frequent and larger blood vessels were recently found in lesions from FOP donors compared with nongenetic forms of HO.^34^ Electron microscopy analysis has revealed that vessels in FOP lesions are usually leaky and hemorrhagic,^35^ with some endothelial cells coexpressing typical osteoblast and fibroblast markers.^18^, ^36^ Considering the tight link between angiogenesis and osteogenesis, ECFCs may be useful to study antiangiogenic therapies in FOP patients, which may provide therapeutic alternatives to prevent HO in FOP.

The recent discovery that activin A induces p‐Smad1 signaling via mutant ALK2, has expanded the potential therapeutic strategies to prevent HO signaling in FOP (reviewed in^37^), including impairment of the activin A‐ALK2 complex formation by means of ligand traps of neutralizing antibodies, genetic downregulation of *ACVR1*, and mutant ALK2 kinase‐activity inhibition. Interestingly, ALK2 was initially identified as an activin receptor,^38^ where it limits the activity of ALK2 BMP ligands through direct competition for the kinase receptor.^39^, ^40^ Therefore, it should be taken into account, however, that activin A antagonists may interfere with the normal physiological functions of the ligand through p‐Smad2/3,^4^ or compromising its BMP counterbalancing effect, potentially leading to undesirable side effects.^41^

On the other hand, because of the high structural similarity among the TGF‐β and BMP receptors, and in particular BMP receptors ALK1, ALK2, and ALK3, to date it has been difficult to obtain molecules or scaffolds with enough ALK2 selectivity to enter in clinical studies. In this regard, macrocyclization has resulted in OD36 and OD52, with enhanced specificity for ALK2. In contrast to the reference compound LDN‐193189, the macrocycles OD36 and OD52 inhibit activin A‐induced p‐Smad1 activity, while having less effect on BMP‐6‐induced downstream signaling. This likely reflects the weaker inhibitory activity of OD36 and OD52 towards ALK3 and ALK6, which may contribute to BMP6 signaling, but not to the activin A‐induced p‐Smad1/5 pathway.

Nowadays there are at least seven therapies under consideration for the treatment of FOP. Two of them, a human specific antiactivin antibody (REGN2477, Regeneron, NCT03188666; Regeneron Pharmaceuticals, Tarrytown, NY, USA) and a RARγ agonist (Palovarotene, NCT02279095; Clementia, Montreal, Canada) are currently in clinical trials. In addition, the mTOR inhibitor rapamycin (Sirolimus(42); Rapamune, Pfizer, New York, NY, USA), the repurposed Src inhibitor anticancer drug saracatinib (AstraZeneca Pharmaceuticals, Cambridge, UK) that also inhibits ALK2 signaling,^9^ an anti‐ALK2 blocking antibody by Daiichi Sankyo (Tokyo, Japan) and Saitama University (Saitama, Japan), and three ALK2 kinase inhibitors (Blueprint Medicines, Cambridge, MA, USA; La Jolla Pharmaceutical Co, San Diego, CA, USA; BioCryst Pharmaceuticals, Durham, NC, USA) have been publicly reported. Apart from waiting for the outcome of clinical trials where periodic systemic administration is considered, it would be of interest to explore whether these drugs may be useful applied locally, eg, surgery to remove heterotopic bone, or for example, in combinations at a lower dose. This might be particularly relevant for drugs inhibiting HO via interfering with different pathways.

In summary, we have introduced and characterized a novel surrogate in vitro model for FOP that we expect to be helpful in the development of new therapeutic approaches for FOP. Overall, our data suggest that ECFCs can be a useful and cost‐effective model system to investigate the cellular and molecular mechanisms underlying FOP with the potential to aid the future identification of predictive flare‐up biomarkers in FOP that are critical for any therapeutic approach investigated. Additionally, the discovery of macrocycles OD36 and OD52 represents a new class of ALK2 inhibitors with enhanced activin‐like receptor kinase selectivity and the potential for reduced off‐target effects. We expect that these encouraging results will contribute to the pursuit of medicinal chemistry efforts towards the identification of a future clinical candidate for FOP.

## Disclosures

The coauthors PB, JC, and JH work for Oncodesign S.A. (Dijon, France), which has developed and patented OD36 and OD52. The other coauthors declare no competing interest.

## Supporting information


**Fig. S1.** A) Western blot showing PDGFRα expression in three independent control and FOP ECFCs. Gene expression analysis of the genes in ECFCs treated with TNF‐α (10 ng/mL) for 24 hours. B) Expression analysis of the genes *FN1* (encoding for Fibronectin), *CDH2* (encoding for N‐Cadherin), *TAGLN* (encoding for SM22α), *CDH5* (encoding for Ve‐Cadherin) and *Pecam‐1*. C) qPCR analysis of the HO progenitor marker *Mx1*. Charts correspond to three independent control and FOP donors. *p < 0.05, **p < 0.01, ***p < 0.001.

**Fig. S2.** Analysis of gene expression by qPCR of the BMP and TGF‐β type I and type II receptors, as well as co‐receptors in ECFC clones from three independent control and FOP donors. *p < 0.05.

**Fig. S3.** BMP‐9 ligand affinity labeling of cell surface receptors performed on murine embryonic 2H11 cells.

**Fig. S4.** A) Chondrogenic differentiation micromass assay in ATDC5 cells over‐expressing ALK2wt or ALK2 R206H. Cells were incubated for 21 days in chondrogenic medium containing BMP‐6 or Activin A (50 ng/mL), prior to Alcian Blue staining. B) Quantification of Fig. A) by absorbance, after solubilization with MetOH. C) Chondrogenic gene expression analysis of *Collagen 2* (Col2), *Sox9*, *Matrix metalloproteinase 13* (Mmp13) and *Collagen X* (Col X) in ATDC5 cells over‐expressing ALK2 R206H and incubated for 21 days in chondrogenic medium containing BMP‐6 or Activin A (50 ng/mL). *p < 0.05, **p < 0.01, ***p < 0.001.

**Table S1.** Clinical aspects of ECFCs donors participating in this study.
**Table S2:** Proqinase Kinase profiling for OD36 and OD52.
**Table S3.** Solubility and metabolic clearance of selected macrocyclic inhibitors.
**Table S4:** Diffraction data collection and refinement statistics.
