# Supplementary figures and images for: Development of Macrocycle Kinase Inhibitors for ALK2 Using Fibrodysplasia Ossificans Progressiva‐Derived Endothelial Cells
Source: JBMR Plus. 2019 Oct 7;3(11):e10230. doi: 10.1002/jbm4.10230 (PMC6874179; doi:10.1002/jbm4.10230)

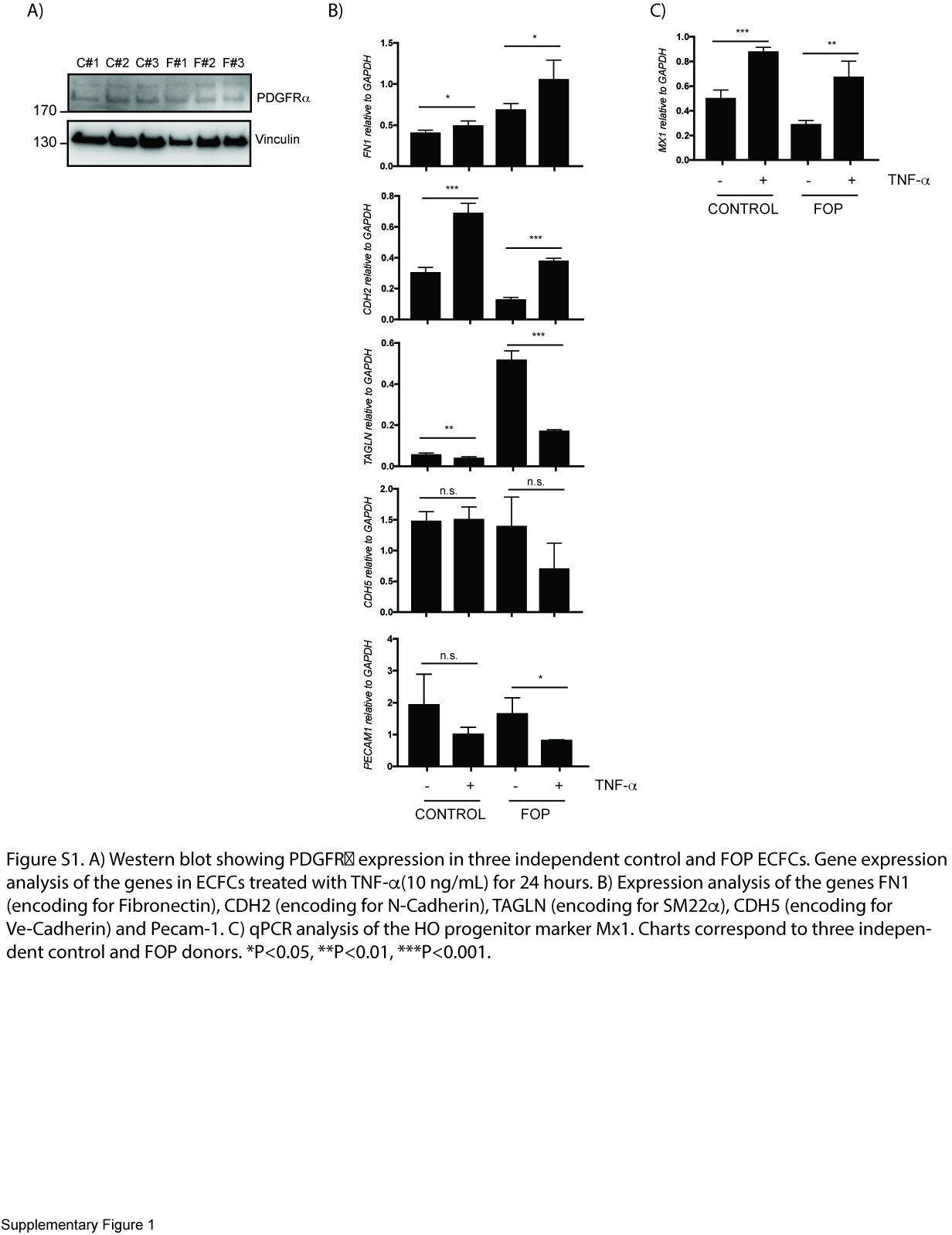

Supplement: Supplementary file 1 — Fig. S1. A) Western blot showing PDGFRα expression in three independent control and FOP ECFCs. Gene expression analysis of the genes in ECFCs treated with TNF‐α (10 ng/mL) for 24 hours. B) Expression analysis of the genes FN1 (encoding for Fibronectin), CDH2 (encoding for N‐Cadherin), TAGLN (encoding for SM22α), CDH5 (encoding for Ve‐Cadherin) and Pecam‐1. C) qPCR analysis of the HO progenitor marker Mx1. Charts correspond to three independent control and FOP donors. *p < 0.05, **p < 0.01, ***p < 0.001. [file JBM4-3-na-s001.tif]

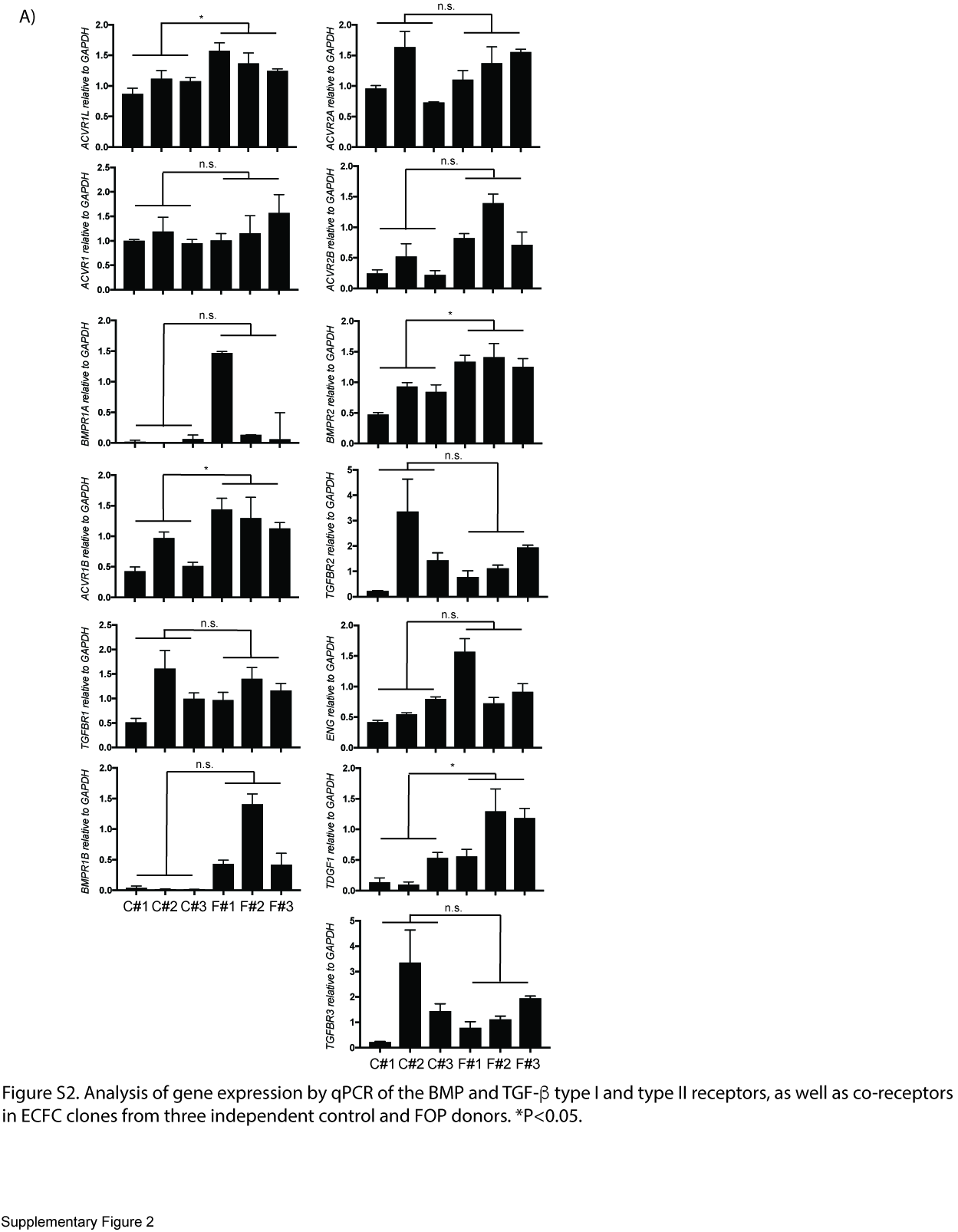

Supplement: Supplementary file 2 — Fig. S2. Analysis of gene expression by qPCR of the BMP and TGF‐β type I and type II receptors, as well as co‐receptors in ECFC clones from three independent control and FOP donors. *p < 0.05. [file JBM4-3-na-s002.tif]

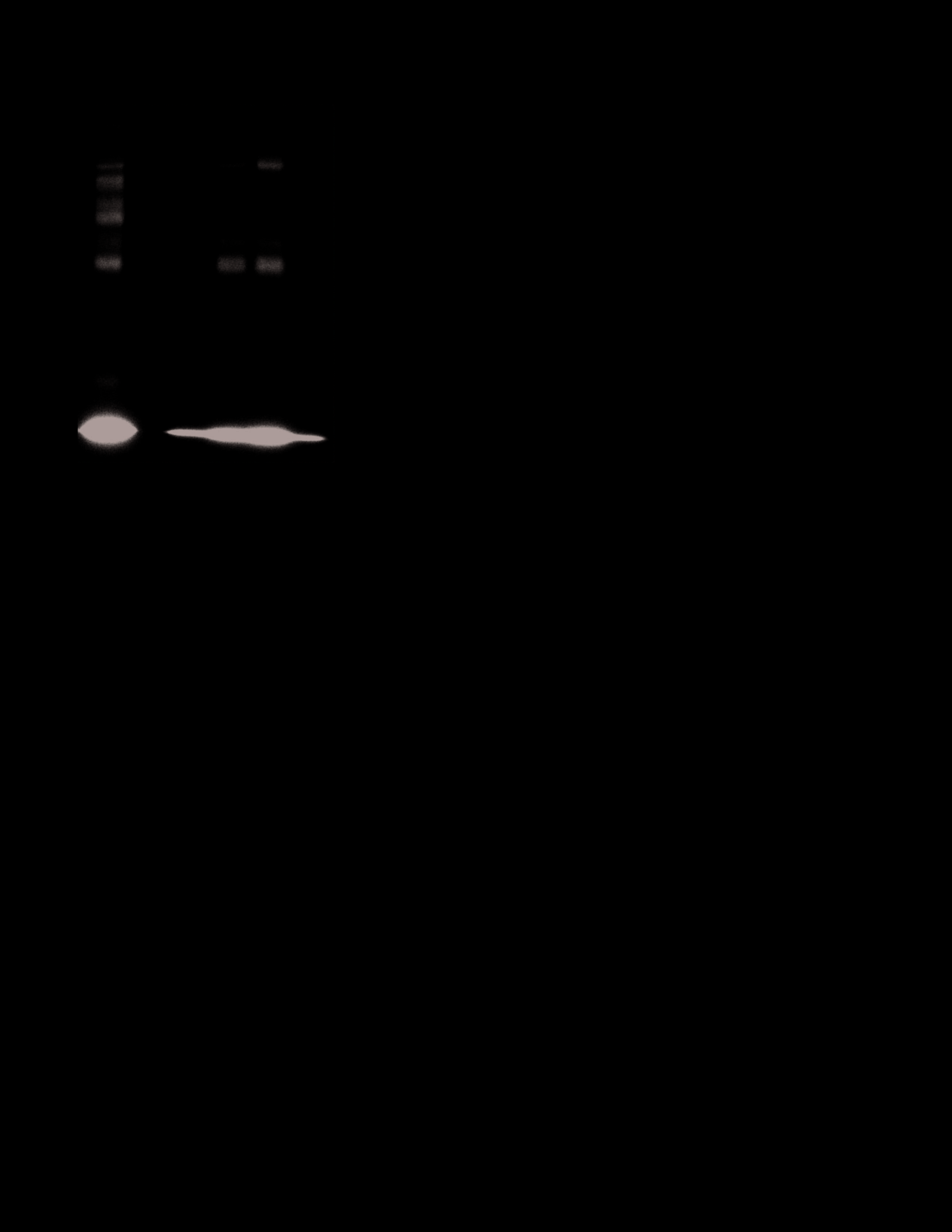

Supplement: Supplementary file 3 — Fig. S3. BMP‐9 ligand affinity labeling of cell surface receptors performed on murine embryonic 2H11 cells. [file JBM4-3-na-s003.tiff]

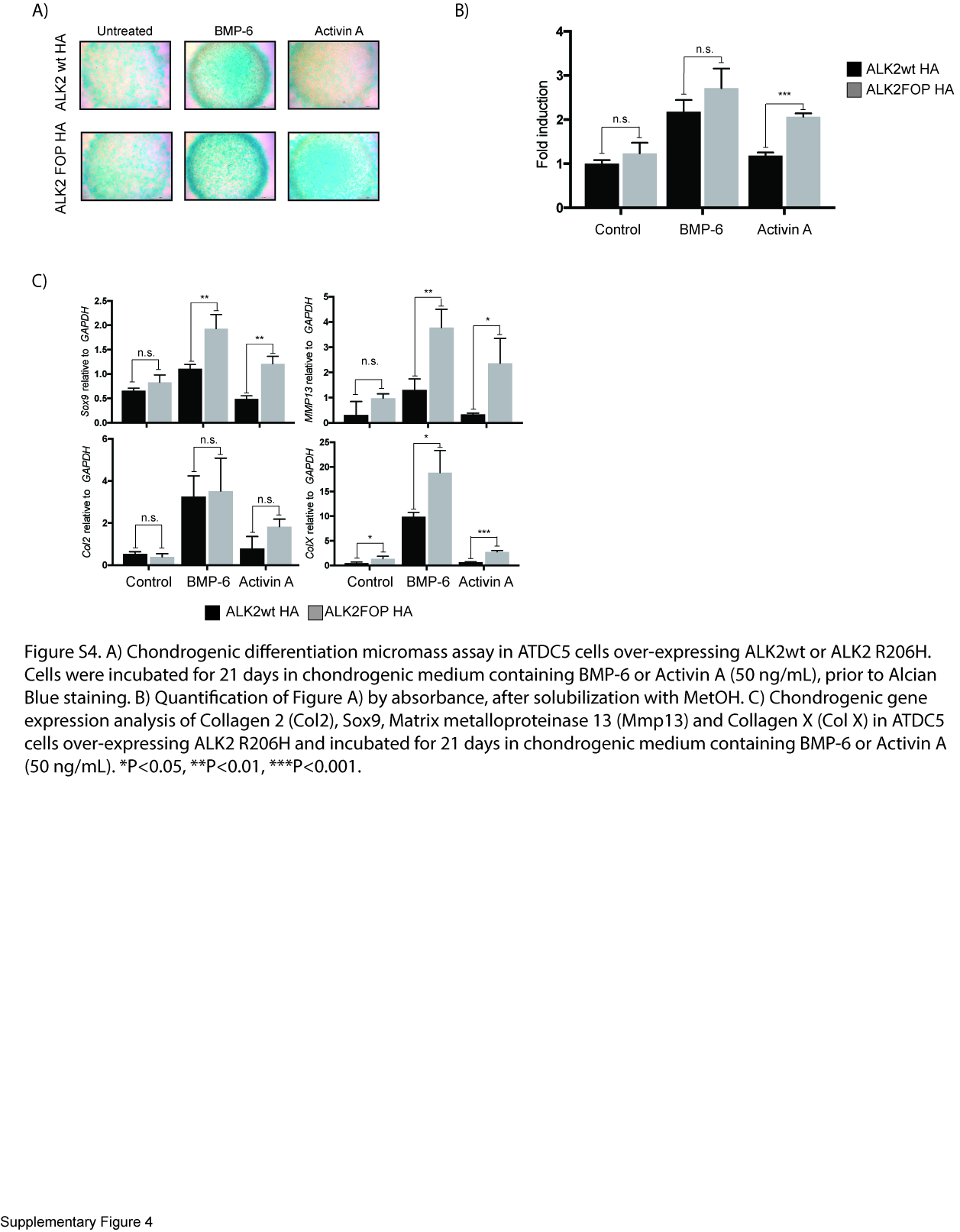

Supplement: Supplementary file 4 — Fig. S4. A) Chondrogenic differentiation micromass assay in ATDC5 cells over‐expressing ALK2wt or ALK2 R206H. Cells were incubated for 21 days in chondrogenic medium containing BMP‐6 or Activin A (50 ng/mL), prior to Alcian Blue staining. B) Quantification of Fig. A) by absorbance, after solubilization with MetOH. C) Chondrogenic gene expression analysis of Collagen 2 (Col2), Sox9, Matrix metalloproteinase 13 (Mmp13) and Collagen X (Col X) in ATDC5 cells over‐expressing ALK2 R206H and incubated for 21 days in chondrogenic medium containing BMP‐6 or Activin A (50 ng/mL). *p < 0.05, **p < 0.01, ***p < 0.001. [file JBM4-3-na-s004.tif]
