# Supplementary material for: Development of Macrocycle Kinase Inhibitors for ALK2 Using Fibrodysplasia Ossificans Progressiva‐Derived Endothelial Cells
Source: JBMR Plus. 2019 Oct 7;3(11):e10230. doi: 10.1002/jbm4.10230 (PMC6874179; doi:10.1002/jbm4.10230)
Supplement: Supplementary file 5 — Table S1. Clinical aspects of ECFCs donors participating in this study. Table S2: Proqinase Kinase profiling for OD36 and OD52. Table S3. Solubility and metabolic clearance of selected macrocyclic inhibitors. Table S4: Diffraction data collection and refinement statistics. [file JBM4-3-na-s005.docx]

**SUPPLEMENTARY FIGURES AND TABLES.**

**Figure S1.** A) Western blot showing PDGFRα expression in three independent control and FOP ECFCs. Gene expression analysis of the genes in ECFCs treated with TNF-α (10 ng/mL) for 24 hours. B) Expression analysis of the genes *FN1* (encoding for Fibronectin), *CDH2* (encoding for N-Cadherin), *TAGLN* (encoding for SM22α), *CDH5* (encoding for Ve-Cadherin) and *Pecam-1*. C) qPCR analysis of the HO progenitor marker *Mx1.* Charts correspond to three independent control and FOP donors. *P<0.05, **P<0.01, ***P<0.001.

**Figure S2.** Analysis of gene expression by qPCR of the BMP and TGF-β type I and type II receptors, as well as co-receptors in ECFC clones from three independent control and FOP donors**.** *P<0.05.

**Figure S3.** BMP-9 ligand affinity labeling of cell surface receptors performed on murine embryonic 2H11 cells.

**Figure S4.** A) Chondrogenic differentiation micromass assay in ATDC5 cells over-expressing ALK2wt or ALK2 R206H. Cells were incubated for 21 days in chondrogenic medium containing BMP-6 or Activin A (50 ng/mL), prior to Alcian Blue staining. B) Quantification of Figure A) by absorbance, after solubilization with MetOH. C) Chondrogenic gene expression analysis of *Collagen 2* (Col2), *Sox9*, *Matrix metalloproteinase 13* (Mmp13) and *Collagen X* (Col X) in ATDC5 cells over-expressing ALK2 R206H and incubated for 21 days in chondrogenic medium containing BMP-6 or Activin A (50 ng/mL). *P<0.05, **P<0.01, ***P<0.001.

**Table S1:** Clinical aspects of ECFCs donors participating in this study.

|  | **Age at biopsy** | **Gender** | **Age at 1^st^ flare-up** | **Flare-ups** | **Medication** |
| --- | --- | --- | --- | --- | --- |
| **FOP#1** | **21,9** | **Female** | **0** | **13** | **Celebrex** |
| **FOP#2** | **23,2** | **Male** | **3** | **2** | **-** |
| **FOP#3** | **24,9** | **Female** | **0** | **10** | **Zometa** |
| **CON#1** | **29,5** | **Female** | **N/A** | **N/A** | **-** |
| **CON#2** | **27,2** | **Male** | **N/A** | **N/A** | **-** |
| **CON#3** | **27,9** | **Female** | **N/A** | **N/A** | **-** |

**Table S2:** Proqinase Kinase profiling for OD36 and OD52.

| **Kinase** | **OD36 % inhibition at 100nM** | **Kinase** | **OD52 % inhibition at 100nM** |
| --- | --- | --- | --- |
| RIPK2 | 97 | ACVR1 | 90 |
| ACVR1 | 95 | ACVRL1 | 89 |
| SIK2 | 94 | RET_S891A | 84 |
| ACVR2B | 90 | RIPK2 | 61 |
| ACVRL1 | 88 | CDK8_cyclinC | 44 |
| ZAK | 70 | LRRK2_G2019S | 39 |
| TGFBR2 | 68 | RET_R813Q | 37 |
| RET_S891A | 63 | RET_R749T | 36 |
| FLT3_D835Y | 62 | PRKX | 34 |
| LRRK2_G2019S | 61 | RET_M918T | 33 |
| DDR2 | 59 | DDR2_N456S | 33 |
| FYN | 59 | RET_Y791F | 30 |
| CDK8_cyclinC | 57 | NTRK1 | 29 |
| PDGFRB | 57 | NTRK2 | 28 |
| LRRK2_R1441C | 56 | RET | 28 |
| LRRK2 | 53 | MAPKAPK5 | 26 |
| GSG2 | 50 | TXK | 25 |
| LRRK2_I2020T | 49 | CAMK2A | 25 |
| LCK | 48 | RET_G691S | 24 |
| HIPK4 | 47 | NTRK3 | 24 |
| PDGFRA_D842V | 47 | LRRK2_I2020T | 22 |
| PTK6 | 46 | SGK1 | 22 |
| RET_R813Q | 45 | CSNK1E | 20 |
| FLT4 | 44 | GSG2 | 20 |
| LYN | 43 | FLT3_ITD | 19 |
| MAP4K4 | 38 | MAP4K5 | 19 |
| TXK | 37 | RET_E762Q | 19 |
| FLT3_ITD | 36 | ACVR2B | 17 |
| KIT_D816V | 36 | WNK2 | 17 |
| GCK | 35 | PDGFRA_D842V | 17 |
| FRK | 35 | MAP2K4 | 17 |
| BLK | 35 | BRAF | 16 |
| KIT | 33 | CSNK1G1 | 16 |
| RET_E762Q | 33 | LRRK2_R1441C | 16 |
| RET_M918T | 33 | PKN3 | 16 |
| ABL1_Q252H | 33 | GRK4 | 16 |
| NLK | 33 | IRAK1 | 16 |
| CAMK2B | 32 | TLK1 | 16 |
| KDR | 32 | MELK | 15 |
| KIT_D816H | 32 | EPHB2 | 15 |
| TLK2 | 31 | DDR2_T654M | 15 |
| MAP3K11 | 30 | ACVR2A | 14 |
| RET_Y791F | 30 | MAP3K11 | 14 |
| RET_R749T | 30 | MAP3K2 | 14 |
| HCK | 29 | ACVR1B | 13 |
| PIM1 | 29 | MAP3K3 | 13 |
| PDGFRA | 29 | STK24 | 12 |
| MKNK2 | 28 | CAMK2B | 12 |
| YES1 | 28 | GRK7 | 12 |
| NTRK2 | 28 | PDGFRA_V561D | 12 |
| RET | 27 | MYLK | 11 |
| PRKCA | 26 | CDC42BPB | 11 |
| FGR | 26 | PRKCG | 11 |
| PIM3 | 25 | DAPK3 | 11 |
| RET_G691S | 25 | TSSK2 | 11 |
| MAPK12 | 24 | LRRK2 | 10 |
| STK17A | 23 | ADRBK1 | 10 |
| MYLK2 | 23 | CAMK2G | 10 |
| MKNK1 | 23 | SLK | 10 |
| EPHA7 | 23 | RPS6KA1 | 10 |
| NEK3 | 22 | AXL | 10 |
| CLK4 | 22 | HIPK4 | 10 |
| ACVR1B | 22 | MAP2K1 | 10 |
| PRKAA1 | 21 | PIM3 | 10 |
| ABL1_H396P | 21 | JAK2 | 9 |
| MELK | 21 | DYRK2 | 9 |
| BRAF | 20 | MKNK2 | 9 |
| FLT1 | 20 | STK16 | 9 |
| CAMKK1 | 20 | TTBK1 | 8 |
| CHUK | 20 | PRKCA | 8 |
| KIT_V560G | 19 | ALK | 8 |
| RPS6KA4 | 19 | STK4 | 8 |
| PBK | 19 | PAK4 | 7 |
| EGFR_G719C | 19 | ABL1_Y253F | 7 |
| MAP4K5 | 18 | ABL1_E255K | 7 |
| PTK2 | 18 | RPS6KA5 | 7 |
| CAMK2A | 18 | MAP2K2 | 7 |
| JAK1 | 18 | DCLK2 | 7 |
| WNK1 | 18 | CLK1 | 6 |
| PAK1 | 18 | CDK1_cyclinB | 6 |
| PLK2 | 17 | DDR2 | 6 |
| EGFR_S752-I759del | 17 | NEK3 | 6 |
| TLK1 | 17 | CDC7_DBF4 | 6 |
| MAPK15 | 17 | ABL1_G250E | 6 |
| DAPK2 | 17 | PKMYT1 | 6 |
| CDK9_cyclinT1 | 17 | MET_D1228N | 6 |
| TNK2 | 17 | VRK2 | 6 |
| PIM2 | 17 | PTK2B | 6 |
| PRKX | 17 | MAP2K6 | 6 |
| NTRK3 | 17 | ZAK | 6 |
| MAPK10 | 17 | INSRR | 6 |
| KIT_A829P | 17 | FYN | 6 |
| CDK9_cyclinK | 16 | NEK7 | 5 |
| AURKB | 16 | PKN1 | 5 |
| LIMK1 | 16 | STK3 | 5 |
| ABL1_M351T | 16 | ROS1 | 5 |
| PRKCG | 16 | CDK2_cyclinE | 5 |
| DAPK3 | 16 | FGFR1 | 5 |
| HIPK2 | 16 | CDK3_cyclinE | 5 |
| GRK4 | 16 | MAP4K4 | 5 |
| CSF1R | 16 | PAK7 | 5 |
| PKN1 | 16 | STK39 | 5 |
| SGK1 | 16 | CSNK1D | 5 |
| KIT_V559D | 15 | CSNK2A2 | 5 |
| TAOK2 | 15 | RPS6KB2 | 4 |
| MERTK | 15 | CSNK1G2 | 4 |
| EGFR_L861Q | 15 | PDGFRA_T674I | 4 |
| MYLK3 | 15 | DNAPK | 4 |
| SRC | 15 | NEK11 | 4 |
| WEE1 | 15 | STK17A | 4 |
| ACVR2A | 15 | FLT3_D835Y | 4 |
| CDC42BPA | 15 | PRKCZ | 4 |
| MAPK13 | 15 | LCK | 4 |
| FGFR1 | 14 | PIM2 | 4 |
| NEK1 | 14 | MAP3K9 | 4 |
| DYRK4 | 14 | PRKD2 | 4 |
| TYK2 | 14 | TTBK2 | 4 |
| FRAP1 | 14 | DYRK4 | 4 |
| PRKG2 | 14 | MAP2K7 | 4 |
| NTRK1 | 14 | PRKD3 | 4 |
| CLK3 | 14 | TGFbR1 | 4 |
| DYRK1B | 13 | PAK6 | 4 |
| ALK_C1156Y | 13 | CLK4 | 4 |
| AURKC | 13 | MAPK13 | 3 |
| MARK4 | 13 | NEK2 | 3 |
| MAP3K9 | 13 | WEE1 | 3 |
| DNAPK | 13 | CSNK2A1 | 3 |
| MAP3K10 | 13 | CLK3 | 3 |
| SLK | 13 | ITK | 3 |
| MAP3K5 | 13 | TAOK2 | 3 |
| ALK_F1174S | 13 | PDGFRB | 3 |
| IRAK4 | 13 | PASK | 3 |
| STK16 | 12 | STK25 | 2 |
| MYLK | 12 | AKT3 | 2 |
| MET_M1250T | 12 | LIMK2 | 2 |
| PRKCQ | 12 | CAMKK2 | 2 |
| MAPKAPK5 | 12 | EIF2AK1 | 2 |
| TEK_Y897S | 12 | CDK6_cyclinD1 | 2 |
| STK3 | 12 | MYLK3 | 2 |
| DAPK1 | 12 | GRK5 | 2 |
| HIPK3 | 12 | CSNK1G3 | 2 |
| MAPK9 | 12 | PRKCD | 2 |
| ERBB4 | 12 | MAPK10 | 1 |
| EPHA3 | 12 | CDK1_cyclinA2 | 1 |
| CDK16_cyclinY | 12 | CLK2 | 1 |
| MAPK3 | 12 | GSK3A | 1 |
| GSK3B | 11 | GCK | 1 |
| PRKCI | 11 | MAPK3 | 1 |
| RPS6KB2 | 11 | ABL1_Q252H | 1 |
| CDK3_cyclinE | 11 | CSNK1A1 | 1 |
| RPS6KA3 | 11 | ROCK1 | 1 |
| SRMS | 11 | NLK | 1 |
| MAPK11 | 11 | MAPK14 | 1 |
| DCLK2 | 11 | CHUK | 0 |
| MARK1 | 11 | RPS6KA6 | 0 |
| CDK1_cyclinA2 | 11 | PTK6 | 0 |
| TBK1 | 11 | CDK2_cyclinA2 | 0 |
| STK4 | 11 | TEC | 0 |
| CHEK1 | 11 | SGK3 | 0 |
| MARK2 | 11 | TGFBR2 | 0 |
| EPHA8 | 11 | DAPK1 | 0 |
| EGFR_L858R | 11 | FGR | 0 |
| EPHA5 | 10 | EGFR_T790M_L858R | 0 |
| CLK2 | 10 | ABL1 | 0 |
| CSNK1D | 10 | HCK | 0 |
| CDK5_p25 | 10 | HIPK1 | 0 |
| MAP3K14 | 10 | TBK1 | -1 |
| EGFR_G719S | 10 | PLK1 | -1 |
| TAOK3 | 10 | PLK4 | -1 |
| MAPK1 | 10 | PAK1 | -1 |
| GRK6 | 10 | CSF1R | -1 |
| EGFR_L747-S752del_P753S | 10 | MET_Y1230A | -1 |
| AXL | 10 | MAPK1 | -1 |
| TTK | 10 | SGK2 | -1 |
| BRAF_V600E | 10 | EIF2AK2 | -1 |
| SRPK1 | 10 | SIK2 | -1 |
| SRPK3 | 9 | EPHA1 | -1 |
| FES | 9 | NEK9 | -1 |
| BMPR1A | 9 | RAF1 | -1 |
| CDK1_cyclinB | 9 | BRAF_V600E | -1 |
| TSSK1B | 9 | BRSK1 | -1 |
| IRAK1 | 9 | PTK2 | -1 |
| PRKD2 | 9 | MARK3 | -1 |
| RPS6KA1 | 9 | DSTYK | -1 |
| EIF2AK2 | 9 | TAOK3 | -1 |
| ABL1_T351I | 9 | FRAP1 | -1 |
| NUAK2 | 9 | MAPKAPK2 | -1 |
| MATK | 9 | ALK_R1275Q | -1 |
| CDK5_p35 | 9 | EPHA5 | -1 |
| EGFR_E746-A750del | 9 | WNK1 | -2 |
| FGFR1_V561M | 9 | CDK16_cyclinY | -2 |
| KIT_T670I | 9 | CDK5_p25 | -2 |
| BMX | 9 | PRKCQ | -2 |
| FGFR4 | 9 | RPS6KA2 | -2 |
| BRSK1 | 8 | IRAK4 | -2 |
| MAP3K1 | 8 | CDK1_cyclinE1 | -2 |
| PKN2 | 8 | RPS6KB1 | -2 |
| MARK3 | 8 | SIK1 | -2 |
| RPS6KA6 | 8 | BTK | -2 |
| CHEK2 | 8 | FGFR3_K650M | -2 |
| PHKG1 | 8 | SYK | -2 |
| ABL1_F317I | 8 | HIPK2 | -2 |
| CSNK2A2 | 8 | NEK4 | -3 |
| FGFR2 | 8 | PKN2 | -3 |
| TEK_R849W | 8 | CHEK2 | -3 |
| HIPK1 | 8 | MAPK15 | -3 |
| MET_D1228H | 8 | ABL1_F317I | -3 |
| PAK7 | 8 | TYK2 | -3 |
| FGFR3_K650E | 8 | ADRBK2 | -3 |
| ALK_F1174L | 8 | MAP3K10 | -3 |
| CDK1_cyclinE1 | 8 | IKBKB | -3 |
| CDK4_cyclinD1 | 8 | CDK9_cyclinT1 | -3 |
| STK24 | 8 | JAK3 | -3 |
| MAP3K2 | 7 | PIM1 | -3 |
| JAK2 | 7 | MARK4 | -4 |
| RPS6KA2 | 7 | PHKG1 | -4 |
| CDK2_cyclinE | 7 | AKT1 | -4 |
| ROCK2 | 7 | NUAK1 | -4 |
| CLK1 | 7 | WNK3 | -4 |
| ROS1 | 7 | NEK1 | -4 |
| EPHA2 | 7 | MYLK2 | -4 |
| DYRK1A | 7 | KIT | -4 |
| MET_Y1230C | 7 | PLK2 | -4 |
| KIT_V559D_T670I | 7 | MET_F1200I | -4 |
| PRKCB1_I | 7 | CDC42BPA | -4 |
| SGK3 | 7 | DYRK1A | -4 |
| CDC42BPB | 7 | AURKB | -4 |
| PLK1 | 7 | INSR | -4 |
| FGFR3 | 7 | CAMK1D | -4 |
| LTK | 6 | FLT1 | -4 |
| GSK3A | 6 | EGFR_L861Q | -4 |
| LIMK2 | 6 | LIMK1 | -5 |
| TEC | 6 | MAPK9 | -5 |
| IKBKE | 6 | TLK2 | -5 |
| JAK3 | 6 | MET | -5 |
| INSR | 6 | ALK/NPM1 | -5 |
| FER | 6 | PRKCB1_I | -5 |
| EPHA1 | 6 | FRK | -5 |
| TEK | 6 | CDK4_cyclinD3 | -5 |
| NUAK1 | 6 | KIT_D816V | -5 |
| ALK | 6 | MAPK11 | -5 |
| FLT3 | 6 | GRK6 | -5 |
| MAP2K1 | 6 | TSSK1B | -5 |
| CAMK2D | 6 | DYRK3 | -5 |
| ALK_R1275Q | 6 | DMPK | -5 |
| CSNK1E | 6 | SRPK3 | -5 |
| EPHB4 | 6 | PBK | -5 |
| SGK2 | 6 | FGFR2 | -6 |
| GRK7 | 6 | ALK_L1196M | -6 |
| CAMK1D | 6 | AURKA | -6 |
| KIT_V654A | 5 | ALK_F1174L | -6 |
| RET_V804M | 5 | CDK7_cyclinH_MAT1 | -6 |
| TYRO3 | 5 | BLK | -6 |
| ABL1_G250E | 5 | ALK_C1156Y | -6 |
| NEK7 | 5 | JAK1 | -6 |
| TGFbR1 | 5 | EPHA8 | -6 |
| WNK3 | 5 | CSK | -6 |
| MAP3K8 | 5 | KIT_V559D | -6 |
| PRKCH | 5 | PRKG1 | -6 |
| MINK1 | 5 | SRC | -6 |
| MET_F1200I | 4 | DYRK1B | -6 |
| PDK1 | 4 | EPHA2 | -6 |
| MET_D1228N | 4 | MAP3K7 | -6 |
| ADRBK1 | 4 | RET_V804M | -6 |
| AKT1 | 4 | PRKCB1_II | -7 |
| NEK11 | 4 | PAK2 | -7 |
| PRKCD | 4 | EPHA4 | -7 |
| CDK2_cyclinA2 | 4 | NUAK2 | -7 |
| MST4 | 3 | ABL1_M351T | -7 |
| ABL1_Y253F | 3 | CDK9_cyclinK | -7 |
| EGFR | 3 | PRKACA | -7 |
| ABL2 | 3 | PRKCE | -7 |
| CSNK1G3 | 3 | FGFR3_K650E | -7 |
| PASK | 3 | TEK | -7 |
| DSTYK | 3 | PDK1 | -7 |
| MET | 3 | BMPR1A | -7 |
| PRKG1 | 3 | TYRO3 | -7 |
| ABL1 | 3 | CDK6_cyclinD3 | -7 |
| BTK | 3 | EPHB3 | -8 |
| MAP3K7 | 3 | KIT_V559D_T670I | -8 |
| CAMKK2 | 3 | CHEK1 | -8 |
| IGF1R | 3 | CDK5_p35 | -8 |
| FGFR3_K650M | 2 | CAMKK1 | -8 |
| DYRK2 | 2 | PLK3 | -8 |
| AKT2 | 2 | RET_V804L | -8 |
| NEK4 | 2 | LTK | -8 |
| EGFR_L747-E749del_A750P | 2 | RPS6KA4 | -8 |
| DMPK | 2 | TTK | -9 |
| RAF1 | 2 | CDK4_cyclinD1 | -9 |
| PRKD3 | 2 | SRMS | -9 |
| IKBKB | 1 | AKT2 | -9 |
| ROCK1 | 1 | MARK2 | -9 |
| MAP3K3 | 1 | EGFR_L747-E749del_A750P | -9 |
| PHKG2 | 1 | EGFR_G719S | -9 |
| MAP2K6 | 1 | MATK | -9 |
| PAK6 | 1 | MAP2K5 | -9 |
| WNK2 | 1 | CAMK2D | -9 |
| STK25 | 1 | ROCK2 | -9 |
| GRK5 | 1 | GSK3B | -9 |
| EIF2AK3 | 1 | KIT_V654A | -9 |
| PAK3 | 1 | FGFR3 | -9 |
| EPHB3 | 1 | SRPK1 | -9 |
| NEK9 | 1 | MARK1 | -9 |
| CSNK1G1 | 1 | MAPK8 | -9 |
| DYRK3 | 0 | FGFR3_G697C | -10 |
| ERBB2 | 0 | MERTK | -10 |
| INSRR | 0 | BUB1B | -10 |
| NEK2 | 0 | MAPK12 | -10 |
| STK39 | 0 | HIPK3 | -10 |
| CDK7_cyclinH_MAT1 | 0 | ERBB4 | -10 |
| ADRBK2 | 0 | PHKG2 | -10 |
| AKT3 | -1 | MAP3K8 | -10 |
| STK33 | -1 | PRKG2 | -10 |
| PDGFRA_V561D | -1 | DAPK2 | -10 |
| PLK4 | -1 | KIT_A829P | -10 |
| PRKCE | -1 | MET_D1228H | -11 |
| SYK | -1 | MKNK1 | -11 |
| MUSK | -1 | AURKC | -11 |
| MAPKAPK3 | -1 | MINK1 | -11 |
| FGFR3_G697C | -1 | MLK4 | -11 |
| SRPK2 | -2 | PRKAA1 | -11 |
| PTK2B | -2 | MUSK | -11 |
| ABL1_E255K | -2 | EGFR_E746-A750del | -11 |
| EPHB1 | -2 | KIT_V559D_V654A | -12 |
| EPHB2 | -2 | PDGFRA | -12 |
| KIT_V559D_V654A | -2 | ABL1_H396P | -12 |
| EPHA4 | -2 | EPHA6 | -12 |
| ZAP70 | -3 | RPS6KA3 | -12 |
| MET_Y1230A | -3 | FER | -12 |
| PAK4 | -3 | MET_Y1230D | -12 |
| MST1R | -3 | MAP3K5 | -12 |
| MET_Y1230H | -3 | NEK6 | -12 |
| CSNK1G2 | -3 | FLT4 | -13 |
| PRKACA | -3 | STK33 | -13 |
| PRKCZ | -3 | ZAP70 | -13 |
| RPS6KA5 | -3 | BRSK2 | -13 |
| CAMK4 | -4 | EEF2K | -13 |
| VRK1 | -4 | MET_Y1230C | -13 |
| PLK3 | -4 | MAP3K1 | -14 |
| EIF2AK1 | -4 | KDR | -14 |
| CSK | -4 | LYN | -14 |
| MAPK14 | -4 | EGFR | -14 |
| CDK6_cyclinD1 | -5 | KIT_D816H | -14 |
| MET_Y1235D | -5 | EIF2AK3 | -14 |
| PAK2 | -5 | VRK1 | -15 |
| PRKCB1_II | -5 | PAK3 | -15 |
| TSSK2 | -6 | EGFR_L747-S752del_P753S | -15 |
| MET_Y1230D | -6 | MET_Y1235D | -15 |
| CSNK1A1 | -6 | EGFR_S752-I759del | -15 |
| ITK | -6 | ABL1_T351I | -15 |
| MAPK8 | -6 | TEK_R849W | -16 |
| AURKA | -7 | SRPK2 | -16 |
| NEK6 | -7 | ALK_F1174L/NPM1 | -16 |
| CSNK2A1 | -7 | MST4 | -17 |
| ALK_L1196M | -7 | PRKCI | -17 |
| MAP2K2 | -7 | EGFR_T790M | -17 |
| RPS6KB1 | -9 | ALK_F1174S | -18 |
| MAPKAPK2 | -9 | FLT3 | -18 |
| EEF2K | -9 | EPHA7 | -18 |
| RET_V804L | -10 | MET_M1250T | -19 |
| PRKD1 | -11 | TNK2 | -20 |
| EGFR_T790M_L858R | -13 | EPHA3 | -20 |
| EGFR_T790M | -14 | PRKCH | -20 |
| PDGFRA_T674I | -22 | MET_Y1230H | -20 |
| TEK_Y1108F | -27 | KIT_V560G | -21 |
| CDK4_cyclinD3 | -30 | IKBKE | -21 |
|  |  | TEK_Y1108F | -21 |
|  |  | PRKD1 | -22 |
|  |  | YES1 | -22 |
|  |  | KIT_T670I | -23 |
|  |  | CAMK4 | -23 |
|  |  | MAP3K14 | -23 |
|  |  | ABL2 | -23 |
|  |  | EGFR_L858R | -23 |
|  |  | MST1R | -24 |
|  |  | EPHB4 | -25 |
|  |  | IGF1R | -25 |
|  |  | MAPKAPK3 | -25 |
|  |  | EPHB1 | -26 |
|  |  | FGFR1_V561M | -26 |
|  |  | FES | -26 |
|  |  | FGFR4 | -28 |
|  |  | EGFR_G719C | -31 |
|  |  | TEK_Y897S | -31 |
|  |  | BMX | -34 |
|  |  | ERBB2 | -44 |
|  |  |  |  |

**Table S3.** Solubility and metabolic clearance of selected macrocyclic inhibitors.

| **ID** | **Chromlog D** | **Solubility in μM** | | **Metabolic clearance (μL/min/mg prot)** | | |
| --- | --- | --- | --- | --- | --- | --- |
|  |  | **PBS** | **FaSSIF** | **mLM** | **rLM** | **hLM** |
| **LDN-193189** | **2,7** | **5** | **49** | **95** | **185** | **61** |
| **OD36** | **5,4** | **2** | **212** | **207** | **74** | **59** |
| **OD52** | **3,9** | **140** | **240** | **84** | **32** | **72** |

**Table S4:** Diffraction data collection and refinement statistics.

|  | **ALK2-OD36** |
| --- | --- |
| Wavelength (Å) | 0.9762 |
| Resolution range (Å)* | 71.9 - 2.56 (2.65 - 2.56) |
| Space group | P 1 2_1_ 1 |
| a, b, c (Å) | 84.26, 103.53, 84.84 |
| α, β, γ (°) | 90.0, 116.5, 90.0 |
| Total reflections* | 81081 (8040) |
| Unique reflections* | 42050 (4176) |
| Multiplicity* | 3.5 (3.6) |
| Completeness (%)* | 99.91 (100.00) |
| Mean I/sigma(I)* | 8.87 (2.34) |
| Wilson B-factor | 63.37 |
| R-merge* | 0.034 (0.251) |
| Reflections used for Refinement | 41945 |
| R-work | 0.2111 |
| R-free | 0.2425 |
| Number of non-hydrogen atoms | 9039 |
| macromolecules | 8868 |
| Ligands | 100 |
| Waters | 71 |
| Protein residues | 1165 |
| RMS(bonds, Å) | 0.007 |
| RMS(angles, °) | 1.17 |
| Ramachandran favored (%) | 97 |
| Ramachandran allowed (%) | 3 |
| Ramachandran outliers (%) | 0 |
| Clashscore | 14.75 |
| Average B-factor, Å^2^ | 88.3 |
| macromolecules | 88.6 |
| ligands | 72.9 |
| solvent | 71.3 |

*Statistics for the highest-resolution shell are shown in parentheses.

**SUPPLEMENTARY MATERIALS AND METHODS**

**Flow cytometric (FACs) analysis of isolated ECFCs**

The combination of the following antibodies was used for the FACs staining: VEC-PE-Cy7 (eBiosciences, 25-1449-41, 1:100), CD31-APC (eBiosciences, 17-0319, 1:100), CD34-APC (Miltenyi Biotech, 130-090-954, 1:20), KDR-PE (R&D Systems, FAB357P, 1:20), CD105-PE (Miltenyi Biotech, 130-098-906, 1:20), CD14-PE (Miltenyi Biotech, 130-091-242, 1:20), CD45-FITC (Miltenyi Biotech, 130-080-202, 1:20). The following isotype matching antibodies were used as a control: IgG1-PE-Cy7 (eBiosciences, 25-4714-41, 1:100), IgG1-PE (R&D Systems, IC002P, 1:20), IgG1-APC (eBiosciences, 17-4714-41, 1:100), IgG2a-PE (R&D Systems, IC003P, 1:20), IgG2a-APC (Miltenyi Biotech, 130-098-850, 1:20). For Tie2 staining non-conjugated antibody were used (Abcam, ab24859, 1ul) followed by secondary Donkey-anti-mouse-A555 (Invitrogen, 1:100).

**Western Blotting**

Cells were seeded into 6-well plates and cultured till they reached confluence. Next, cells were stimulated as indicated and then washed with cold PBS. Lysates were prepared using 2x Sample buffer (10% SDS, 62.5 mM Tris-HCl pH 6.8, 0.002 % Bromophenol Blue, 0.7135 M (5%) β-mercaptoethanol, 10 % glycerol) and boiled for 5 minutes before loading. Electrophoresis was performed in 10% SDS/polyacrylamide gels. Separated proteins were transferred to nitrocellulose membranes (0.5 A at 100 V; in ice) for 1 hour. Blots were blocked in Tris-Buffered Saline solution containing 0.1% Tween 20 and 5% non-fat dry milk overnight at 4 ºC, and immune-detection of specific proteins was carried out with primary antibodies (see above) followed by horseradish peroxidase-conjugated anti-mouse or anti-rabbit (GE Healthcare, Eindhoven, The Netherlands) secondary antibodies using an ECL system (Fisher Scientific, Landsmeer, The Netherlands). A list of the antibodies used can be found below:

| **Antibody** | **Concentration** | **Provider** | **Reference** |
| --- | --- | --- | --- |
| p-Smad1/5 | 1:1000 | Homemade | See ^(1)^ |
| Smad1 | 1:1000 | Santa Cruz | Sc-7965 |
| p-Smad2 | 1:1000 | Homemade | See ^(1)^ |
| Smad2 | 1:1000 | BD Laboratories | BD610842 |
| Gapdh | 1:10000 | Millipore | 6C5 |
| PDGFRα | 1:1000 | Cell Signaling | #3174 |
| Vinculin | 1:1000 | Sigma-Aldrich | V9131 |

**Immunofluorescent labelling**

For immuno-fluorescent labeling of ECFCs *in vitro*, cells grown on coverslips were fixed with 4% formaldehyde for 30 minutes at room temperature, washed with glycine for 5 minutes, permeabilized with 0.2% Triton X-100 and blocked in PSB containing 5% BSA for one hour. Next, the cells were incubated overnight at 4C in blocking solution containing primary antibody with gentle shaking. Next day, the cells were washed 5 times in washing buffer (PBS containing 0.05% Tween-20 and 1% BSA) and incubated with secondary antibody (Alexa Fluor FITC goat anti-mouse IgG, Alexa-Fluor 555 anti-rabbit IgG (Invitrogen, Breda, the Netherlands), or Cy3 goat anti-mouse IgG (Jackson ImmunoResearch, Suffolk, England; 1:200) or Phalloidin-488 (1:100) in PBS with 0.5% BSA for one hour. Finally, the cells were washed 5 times in washing buffer and mounted in Prolong Gold containing DAPI (Invitrogen). After careful drying, the preparations were imaged in a Leica SP5 confocal scanning laser microscope. A representative picture from each staining is shown (n = 3). The following antibodies were used for immunofluorescent staining: Ve-Cadherin (Cell Signaling, #2158, 1:100), Fibronectin (Sigma-Aldrich, F7387, 1:400), Transgelin (SM22α, abcam, ab14106, 1:400), N-Cadherin (BD Transduction Laboratories, 610920, 1:50).

**Quantitative Real Time RT-PCR (qPCR)**

A list of the oligonucleotides used for qPCR can be found below:

hACVR1L forward, 5′- CTGGTTCCGGGAGACTGAGAT-3′

hACVR1L reverse, 5′- TGCGGGAGGTCATGTCTGA-3′

hACVR1 forward, 5′- TTTGAACGCTGCTTGCATGG-3′

hACVR1 reverse, 5′- AGGCTCTTGGTCACATCTGC-3′

hBMPR1A forward, 5′- GGGGTCCGACTTATGAAA-3′

hBMPR1A reverse, 5′- TACGACTCCTCCAAGATGTGG-3′

hACVR1B forward, 5′- CACCTCAGGGTCTGGCTC-3′

hACVR1B reverse, 5′- AACCAAGACCGTTCTTCACG-3′

hTGFBR1 forward, 5′- ACGGCGTTACAGTGTTTCTG-3′

h TGFBR1 reverse, 5′- GCACATACAAACGGCCTATCT-3′

hBMPR1B forward, 5′- GCTCAGGAAGTGGATCAGG-3′

hBMPR1B reverse, 5′- CATGCCTCATCAACACTGTC-3′

hACVR1C forward, 5′- CGCACTTCAAAAGGGTGTCG-3′

hACVR1C reverse, 5′- TGATGCCCAACATGCTCCTT-3′

hBMPR2 forward, 5′- AACTGTTGGAGCTGATTGGC-3′

hBMPR2 reverse, 5′- CGGTTTGCAAAGGGAAAACAC-3′

hACVR2A forward, 5′- CTGCTGCAAAGTTGGCG-3′

hACVR2A reverse, 5′- TGATGCCCAACATGCTCCTT-3′

hACVR2B forward, 5′- ATGTGGACATCCATGAGGAC-3′

hACVR2B reverse, 5′- TGAAGATCCCGTTCACTC-3′

hTBR2 forward, 5′- GCAGGTGGGAACTGCAAGAT-3′

hTBR2 reverse, 5′- GAAGGACTCAACATTCTCCAAATTC-3′

hENG forward, 5′- CCGAGAGGTGCTTCTGGTCC-3′

hENG reverse, 5′- GTGCAGTGGGATTCCCAGG-3′

hTDGF1 forward, 5′- CACGATGTGCGCAAAGAGAA-3’

hTDGF1 reverse, 5′- TGACCGTGCCAGCATTTACA-3′

hTBR3 forward, 5′- GCTGACGCTGTGTACGAAG-3′

hTBR3 reverse, 5′- TCTGCTTCATGGTGGATCAC-3′

hCol1a1 forward, 5′- AAAGGCAATGCTCAAACACC -3′

hCol1a1 reverse, 5′- TCAAAAACGAAGGGGAGATG -3′

hALP forward, 5′- GACCCTTGACCCCCACAAT-3′

hALP reverse, 5′- GCTCGTACTGCATGTCCCCT-3′

hRunX2 forward, 5′- TGGTTACTGTCATGGCGGGTA -3′

hRunX2 reverse, 5′- TCTCAGATCGTTGAACCTTGCTA -3′

hOsterix forward, 5′- GCCAGAAGCTGTGAAACCTC-3′

hOsterix reverse, 5′- GCCAGAAGCTGTGAAACCTC -3′

hID-1 forward, 5′- TGATGCCCAACATGCTCCTT-3′

hID-1 reverse, 5′- GAAGGTCCCTGATGTAGTCGAT-3′

hID-3 forward, 5′- CACCTCCAGAACGCAGGTGCTG-3′

hID-3 reverse, 5′- AGGGCGAAGTTGGGGCCCAT-3′

hGAPDH forward, 5′-TGCACCACCAACTGCTTAGC-3′

hGAPDH reverse, 5′-GGCATGGACTGTGGTCATGAG-3′

hMx1 forward, 5′- ACCGAAACTGAATTGTCCGGG-3′

hMx1 reverse, 5′- TCCTGCACTGGGCTTACTTC-3′

hScx forward, 5′- GACCGCAAGCTCTCCAAGAT-3′

hScx reverse, 5′- GCGGTCCTTGCTCAACTTTC-3′

hFN1 forward, 5′- CGTCATAGTGGAGGCACTGA-3′

hFN1 reverse, 5′- CAGACATTCGTTCCCACTCA-3′

hTAGLN forward, 5′- CATCCTGTCTGTCCGAACCC-3′

hTAGLN reverse, 5′- GACTGAGAGGGTGGGTTTCC-3′

hCDH5 forward, 5′- TCGTTGCGCTCTTCGTGAC-3′

hCDH5 reverse, 5′- CAGCCCGCAAAACAGGTAG-3′

hCDH2 forward, 5′- CAGACCGACCCAAACAGCAAC-3′

hCDH2 reverse, 5′- GCAGCAACAGTAAGGACAAACATC-3′

hPECAM1 forward, 5′- ATCGGTTGTTCAATGCGTCC-3′

hPECAM1 reverse, 5′- CCTTCAGGATTTGGTACATGACA-3′

mGAPDH forward, 5′- TGGCAAAGTGGAGATTGTTGCC-3′

mGAPDH reverse, 5′- AAGATGGTGATGGGCTTCCCG-3′

mMmp13 forward, 5′- ACCTTGTGTTTGCAGAGCACTAACTT-3′

mMmp13 reverse, 5′- CTTCAGGATTCCCGCAAGAGT-3′

mSox9 forward, 5′- TTCATGAAGATGACCGACGA-3′

mSox9 reverse, 5′- ATGCACACGGGGAACTTATC-3′

mCol2 forward, 5′- TTCCACTTCAGCTATGGCGA-3′

mCol2 reverse, 5′- GACGTTAGCGGTGTTGGGAG-3′

mColXa1 forward, 5′- GCAGCATTACGACCCAAGATC-3′

mColXa1 reverse, 5′- TCTGTGAGCTCCATGATTGC-3′

**Transfections, luciferase assays and DNA constructs.**

For luciferase reporter assays, cells were seeded in 24-well plates and transfected with DharmaFECT Duo (Fisher Scientific, Landsmeer, The Netherlands), following the recommendations of the manufacturer. 48 hours after transfection the cells were harvested and lysed. Luciferase activity was measured using the luciferase reporter assay system from Promega (Leiden, The Netherlands) by a Perkin Elmer luminometer Victor^3^ 1420. Each transfection mixture was equalized with empty vector when necessary and every experiment was performed in triplicate. The BRE-Luc ^(2)^, the CAGA ^(3)^ reporter constructs and the expression vectors for constitutively active ALKs ^(4)^ or the R206H ALK2 construct ^(5)^ have been reported elsewhere.

**Iodination and ligand affinity labeling of cell surface receptors.**

Iodination of BMP9 was performed according to the chloramine T method and cells were subsequently affinity labelled with the radioactive ligand as described before ^(6)^. Cell lysates were immunoprecipitated with specific antibodies against ALK1, ALK2, BMPR2 and ActR2A. The generation and characterization of these antibodies have been previously published ^(7)^.

**Kinome profiling and biochemical data.**

DiscoverX Kd determination: For most assays, kinase-tagged T7 phage strains were prepared in an *E. coli* host derived from the BL21 strain. *E. coli* were grown to log-phase and infected with T7 phage and incubated with shaking at 32°C until lysis. The lysates were centrifuged and filtered to remove cell debris. The remaining kinases were produced in HEK-293 cells and subsequently tagged with DNA for qPCR detection. Streptavidin-coated magnetic beads were treated with biotinylated small molecule ligands for 30 minutes at room temperature to generate affinity resins for kinase assays. The liganded beads were blocked with excess biotin and washed with blocking buffer (SeaBlock (Pierce), 1% BSA, 0.05% Tween 20, 1 mM DTT) to remove unbound ligand and to reduce non-specific binding. Binding reactions were assembled by combining kinases, liganded affinity beads, and test compounds in 1x binding buffer (20% SeaBlock, 0.17x PBS, 0.05% Tween 20, 6 mM DTT). Test compounds were prepared as 111X stocks in 100% DMSO. Kds were determined using an 11-point 3-fold compound dilution series with three DMSO control points. All compounds for Kd measurements are distributed by acoustic transfer (non-contact dispensing) in 100% DMSO. The compounds were then diluted directly into the assays such that the final concentration of DMSO was 0.9%. All reactions performed in polypropylene 384-well plate. Each was a final volume of 0.02 ml. The assay plates were incubated at room temperature with shaking for 1 hour and the affinity beads were washed with wash buffer (1x PBS, 0.05% Tween 20). The beads were then re-suspended in elution buffer (1x PBS, 0.05% Tween 20, 0.5 μM non-biotinylated affinity ligand) and incubated at room temperature with shaking for 30 minutes. The kinase concentration in the eluates was measured by qPCR. An 11-point 3-fold serial dilution of each test compound was prepared in 100% DMSO at 100x final test concentration and subsequently diluted to 1x in the assay (final DMSO concentration = 1%). Most Kds were determined using a compound top concentration = 30.000 nM. If the initial Kd determined was < 0.5 nM (the lowest concentration tested), the measurement was repeated with a serial dilution starting at a lower top concentration. A Kd value reported as 40.000 nM indicates that the Kd was determined to be >30.000 nM. Binding constants (Kds) were calculated with a standard dose-response curve using the Hill equation: Response = Background + Signal – Background 1 + (KdHill Slope / DoseHill Slope). The Hill Slope was set to -1. Curves were fitted using a non-linear least square fit with the Levenberg-Marquardt algorithm.

RBC HotSpot Kinase assay protocol and IC50 determonation: First, the substrate was prepared in freshly prepared Reaction Buffer containing 20 mM Hepes (pH 7.5), 10 mM MgCl_2_, 1 mM EGTA, 0.02% Brij35, 0.02 mg/ml BSA, 0.1 mM Na_3_VO_4_, 2 mM DTT, 1% DMSO. In addition, required cofactors are added individually to each kinase reaction. Next, the ALK2 wt or R206H kinase was delivered kinase into the substrate solution and gently mixed, followed by the compounds (LDN-193189, OD36 or OD52) in 100% DMSO by Acoustic technology (Echo550; nanoliter range). After 20 mins incubation at room temperature, ^33^P-ATP (Specific activity 10 μCi/μl) was added to the reaction mixture to initiate the reaction and incubated for 2 hours at room temperature. Finally, radioactivity was detected by filter-binding method. The kinase activity data were expressed as the percent remaining kinase activity in test samples compared to vehicle (dimethyl sulfoxide) reactions. IC50 values and curve fits were obtained using Prism (GraphPad Software). Km ATP: 5 μM (ALK2 wt); 10 μM ALK2 R206H.

**Microsomal Incubations.**

Pooled human liver microsomes were purchased from Xenotech LLC (Kansas City, KS). Microsomes (0.5 mg/ml) were preincubated with 0.5 μM test compound for 7 min at 37°C in 50 mM phosphate buffer, pH 7.4. The reactions were initiated by adding prewarmed cofactor (0.44 mM NADPH). Aliquots of samples were taken at 0, 5, 10, 20 and 45 min and the reactions terminated by addition of cold acetonitrile containing an internal standard. Samples were centrifuged at 3000g for 10 min at 4°C and then supernatants were analyzed by LC/MS/MS for the amount of parent compound remaining.

**Solubility determination.**

Test compound from 5 mM DMSO stock solution were diluted to 250 µM either in 50 mM PBS pH 7.4 or FaSSIF pH 6.5 in triplicate. DMSO final concentration was 5%. After a 2-hour equilibrium at room temperature and under shaking, samples were centrifuged at 3000g for 10 min at room temperature. A calibration curve was prepared in LC mobile phase from the 5 mM DMSO stock solution. Samples and calibration curve were analyzed by LC with UV detection.

**X-ray crystallography and molecular modelling**

The kinase domain of caALK2 (residues 201–499; Q207D) was cloned into pFB-LIC-Bse for baculoviral expression in Sf9 cells. ALK2 was purified by nickel affinity and size exclusion chromatography. The eluted protein was buffered in 50 mM HEPES pH 7.5, 300 mM NaCl, 2 mM DTT, 50 mM arginine, 50 mM glutamate. The hexahistidine tag was cleaved using tobacco etch virus protease. Crystallization was achieved at 4 °C using the sitting-drop vapor diffusion method. ALK2 was preincubated with 1 mM OD36 at a protein concentration of 13.6 mg/mL and crystallized using a precipitant containing 0.3 M magnesium formate, 0.1 M Bis-Tris pH 5.5. Viable crystals were obtained when the protein solution was mixed with the reservoir solution at 2:1 volume ratio. Crystals were cryoprotected with mother liquor plus 25% ethylene glycol prior to vitrification in liquid nitrogen. Diffraction data were collected at the Diamond Light Source station I04, processed with MOSFLM ^(8)^ and subsequently scaled using AIMLESS from the CCP4 suite ^(9)^. Initial phases were obtained by molecular replacement using PHASER ^(10)^ and the structure of ALK2 (3H9R) as a search model. The resulting structure solution was refined using REFMAC5 from the CCP4 suite ^(11)^, Phenix Refine ^(12)^ and manually rebuilt with COOT ^(13)^. Appropriate TLS restrained refinement using the tls tensor files calculated from the program TLSMD ^(14)^ was applied at the final round of refinement. The complete structure was verified for geometric correctness with MolProbity ^(15)^ and deposited in the Protein Data Bank (PDB 5OY6). Data collection and refinement statistics are shown in Table S4. OD52 was modeled using X-ray informations from the co-crystallographic structure of OD36 in ALK2 with the use of MOE as modeling tool.

**SUPPLEMENTARY REFERENCES.**

1. Nakao A, Imamura T, Souchelnytskyi S, Kawabata M, Ishisaki A, Oeda E, et al. TGF-beta receptor-mediated signalling through Smad2, Smad3 and Smad4. EMBO J. 1997;16(17):5353-62.

2. Korchynskyi O, ten Dijke P. Identification and functional characterization of distinct critically important bone morphogenetic protein-specific response elements in the Id1 promoter. J Biol Chem. 2002;277(7):4883-91.

3. Dennler S, Itoh S, Vivien D, ten Dijke P, Huet S, Gauthier JM. Direct binding of Smad3 and Smad4 to critical TGF beta-inducible elements in the promoter of human plasminogen activator inhibitor-type 1 gene. EMBO J. 1998;17(11):3091-100.

4. Petersen M, Thorikay M, Deckers M, van Dinther M, Grygielko ET, Gellibert F, et al. Oral administration of GW788388, an inhibitor of TGF-beta type I and II receptor kinases, decreases renal fibrosis. Kidney Int. 2008;73(6):705-15.

5. van Dinther M, Visser N, de Gorter DJ, Doorn J, Goumans MJ, de Boer J, et al. ALK2 R206H mutation linked to fibrodysplasia ossificans progressiva confers constitutive activity to the BMP type I receptor and sensitizes mesenchymal cells to BMP-induced osteoblast differentiation and bone formation. J Bone Miner Res. 2010;25(6):1208-15.

6. Scharpfenecker M, van Dinther M, Liu Z, van Bezooijen RL, Zhao Q, Pukac L, et al. BMP-9 signals via ALK1 and inhibits bFGF-induced endothelial cell proliferation and VEGF-stimulated angiogenesis. J Cell Sci. 2007;120(6):964-72.

7. Rosenzweig BL, Imamura T, Okadome T, Cox GN, Yamashita H, ten Dijke P, et al. Cloning and characterization of a human type II receptor for bone morphogenetic proteins. Proc Natl Acad Sci U S A. 1995;92(17):7632-6.

8. Leslie AG. The integration of macromolecular diffraction data. Acta Crystallogr D Biol Crystallogr. 2006;62(1):48-57.

9. Winn MD, Ballard CC, Cowtan KD, Dodson EJ, Emsley P, Evans PR, et al. Overview of the CCP4 suite and current developments. Acta Crystallogr D Biol Crystallogr. 2011;67(4):235-42.

10. McCoy AJ, Grosse-Kunstleve RW, Adams PD, Winn MD, Storoni LC, Read RJ. Phaser crystallographic software. J Appl Crystallogr. 2007;40(4):658-74.

11. Murshudov GN, Vagin AA, Dodson EJ. Refinement of macromolecular structures by the maximum-likelihood method. Acta Crystallogr D Biol Crystallogr. 1997;53(3):240-55.

12. Adams PD, Afonine PV, Bunkoczi G, Chen VB, Davis IW, Echols N, et al. PHENIX: a comprehensive Python-based system for macromolecular structure solution. Acta Crystallogr D Biol Crystallogr. 2010;66(2):213-21.

13. Emsley P, Cowtan K. Coot: model-building tools for molecular graphics. Acta Crystallogr D Biol Crystallogr. 2004;60(12):2126-32.

14. Painter J, Merritt EA. Optimal description of a protein structure in terms of multiple groups undergoing TLS motion. Acta Crystallogr D Biol Crystallogr. 2006;62(4):439-50.

15. Davis IW, Leaver-Fay A, Chen VB, Block JN, Kapral GJ, Wang X, et al. MolProbity: all-atom contacts and structure validation for proteins and nucleic acids. Nucleic Acids Res. 2007;35:W375-83.
